# Mapping What Works to Strengthen the Relational Ecology of Early Child Development: A Systematic Scoping Review

**DOI:** 10.1007/s10567-026-00558-6

**Published:** 2026-04-17

**Authors:** Matthew Fuller-Tyszkiewicz, Suzanne Vassallo, Tracy Evans-Whipp, Kayla Mansour, Gessica Misuraca, Georgia Zoumboulis, Louise Newman, Craig A. Olsson, the Australian Early Relational Health Network, Jacqueline Allen, Cath Chamberlain, Juli Coffin, Donna Cross, Tracy Evans-Whipp, Alex Fischer, Jacinta Francis, Matthew Fuller-Tyszkiewicz, Rebecca Glauert, Melissa Green, Ross Homel, Primrose Letcher, Jacqui A. Macdonald, Kayla Mansour, Jennifer McIntosh, Shaun McLaws, Siobhan M. O’Dean, Craig Olsson, Felicity Painter, Natasha Pearce, Naomi Priest, Lisa Ritland, Tim Slade, Liz Spry, Sarah Whittle, Lu Zhang, Stephen R. Zubrick

**Affiliations:** 1 Faculty of Health, School of Psychology, SEED Lifespan Strategic Research Centre, Deakin University, 221 Burwood Highway, Burwood, VC 3125 Australia; 2 Centre for Adolescent Health, Murdoch Children’s Research Institute, Melbourne Royal Children’s Hospital, Parkville, VC Australia; 3 Department of Paediatrics, The University of Melbourne, Melbourne Royal Children’s Hospital, Parkville, VC Australia; 4 Department of Psychiatry, The University of Melbourne, Parkville, Australia; 5 Griffith University, Brisbane, Australia; 6 The University of Melbourne, Parkville, Australia; 7 Murdoch University, Murdoch, Australia; 8 University of Western Australia, Perth, Australia; 9 Deakin University, Burwood, Australia; 10 Australian National University, Canberra, Australia; 11 University of New South Wales, Kensington, Australia; 12 La Trobe University, Melbourne, Australia; 13 University of Sydney, Camperdown, Australia; 14 Telethon Kids, Nedlands, Australia; 15 University of British Columbia, Vancouver, Canada; 16 The University of Sydney, Camperdown, Australia

**Keywords:** Universal interventions, Relational health, RCT evaluated programs, Scoping review

## Abstract

The development of secure bonds between children and adult carers, particularly parents, is determined by a complex ecosystem of relationships that includes parents, care providers in other settings, and the values and institutions that care for those who care for children. Here we conduct a scoping review to map scientific progress in the development of evidence based interventions across all levels of relational ecology of early child development, form families to communities, the connections between them, and the structures that support them. A systematic search for relevant studies was conducted across three databases: Ovid MEDLINE, PsychInfo, and Embase. A total of 4970 articles were retrieved, with a final retained set of 123 papers (30207 families across 110 unique interventions). Most intervention development has focused on the relationship between children and their mothers. Few interventions directly targeted father involvement in parenting, or broader systems of care within the family (e.g., grandparents) and beyond (e.g., early childcare workers, family friendly workplaces, and the healthcare system). Many population-based programs were situated within existing universal healthcare services for parents of newborns, highlighting scalability and potential for delivering programs proportionate to need. However, further work is needed to convert this potential scalability into a reality. Further investment in intervention development in relational systems beyond the family is urgently needed if we are to create the broader conditions needed to ensure that emotional bonds between children and their adult carers can flourish.

**Supplementary Information:**

The online version contains supplementary material available at 10.1007/s10567-026-00558-6.

## Introduction

The importance of promoting early bonds of trust between children and the adults that care for them (i.e., relational health), particularly within the immediate family system, is well established (Groh et al., 2017; Ranson & Urichuk, 2008; Spruit et al., 2020). Relational difficulties (as the opposite of relational health) have been linked to a wider range of adverse outcomes including later chronic health conditions, psychological developmental delays, and emotional and mental disorder, and the level of educational attainment and employment prospects in adulthood (Moore et al., 2017). Such consequences create ethical, economic, and humanistic imperatives to identify the earliest opportunities to promote relational health from early life in ways that strengthen relational health in families and, in turn, set the foundations for relationally well populations into the future. This necessitates population approaches to strengthening the relational ecology within which early child development happens, from families to communities, the connections between them, and the structures that support them. This systematic scoping review maps scientific progress in the development of evidence-based interventions across all levels of the relational ecology of early child development. Our aim in doing so is to describe what we know, what we don’t know, and what still needs to be done to enable a system of interventions to be deployed to strengthen the relational ecosystem around the developing child.

This complex and multifaceted ecosystem around the developing child has been described in detail within the bioecological model of human development (Bronfenbrenner, 2005). Within this model, much of development proceeds as a function of proximal interactions that happen between children and their parents (biological or other). The motivation for parents to care for their offspring and for offspring to seek out their parents for protection has been central to the development of our species (and many other) for millennia. This protective relational shield is further strengthened by other adult carers, in other settings, within kin and non-kin social networks (e.g., Riem et al., 2023; Sarkadi et al., 2007). It is also strengthened through structures of society that ensure that those who are caring for children are themselves being adequately cared for. Institutional structures include supportive work environments that enable time for child-parent interactions, quality early childhood and education services, community programs that support family life, and social norms that value children and their carers (Bronfenbrenner, 2005; Moore et al., 2017).

The quality of this relational ecosystem determines the overall conditions within which the bonds between children and their adult carers, in whatever settings, become enabled in ways that nurture healthy social, emotional, and cognitive child development (Moore et al., 2017). This highlights the importance of investing across the relational ecology of early childhood with the specific aims of both promoting the conditions within which secure bonds emerge and preventing the conditions within which insecure bonds emerge, in particular those involving early conflict, trauma, and neglect. Investing in universal interventions that strengthen the relational ecology of early child development, particularly if delivered proportionate to need (Marmot et al., 2010), can also be more cost effective than intervening later when more complex relational problems inevitably emerge (Abou Jaoude et al., 2024; Greenberg & Abenavolli, 2016).

Here we present findings from an initial systematic scoping review designed to map scientific progress in developing and evaluating universal (population based) interventions that strengthen the relational ecology of early child development (up to three years of age). We looked for interventions at all levels of the social ecology of early development, from families to early childcare to communities and wider government and non-government social supports. We included population interventions with a focus on relational health outcomes, including attachment, bonding, sensitivity, and parenting with the aim of describing breadth of interventions, level of efficacy, and potential for scale-up, consistent with a proportionate universal approach.

## Method

A scoping review was selected as the preferred methodology for this study as we wished to examine a wide range of interventions in which early relational health is conceptualised and measured in a range of different ways. We extend this methodology to include a narrative synthesis of study findings that helps identify research gaps that may be prioritised for further research and policy work.

### Search Strategy

This review was guided by the Joanna Briggs Institute (JBI) approach to scoping reviews (Peters et al., 2020) and follows the PRISMA (Preferred Reporting Items for Systematic Reviews—Extension for Scoping Reviews; Tricco et al., 2018) guidelines for conduct and reporting of scoping reviews. Three databases (Medline, PsychInfo, and Embase) were searched in July 2023 and an updated search was performed in August 2025. Three concepts were used for this search strategy: (1) child and family relational ecology, reflecting child exposure to relationships and observations of relationships with and between parents, siblings, grandparents, peers and caregivers; (2) first three years of life, reflecting the period from conception up to three years post-partum, and (3) study design, focusing on RCTs (see Online Resource 1 for all terms used for each concept). Searches were limited to journal articles, written in the English language, and describing research involving human subjects. No date limits were applied for this search.

### Eligibility Criteria

Studies were included if they: (1) were written in English; (2) reported on studies of human subjects; (3) involved an RCT study design; (4) reported on a program or intervention that is delivered—or has the potential to be delivered—universally to the population of parents; (5) reported at least one relational health outcome (attachment, bonding, relationship quality, family function, etc.) between child and parent, parent and parent, child (or parent) and grandparent, or child and sibling; and (6) at least one relational health outcome measure was assessed between pregnancy and child age three years.

RCTs were included regardless of whether the comparison was to a wait-list control, attention control, treatment as usual, or other intervention. Studies were excluded if the program or intervention was delivered in a clinical setting and/or targeted a specific/indicated population group based on clinical criteria of relational rupture between infant-parent or other relationships around the child (e.g., foster or adoptive parents, children of parents experiencing domestic violence, substance use, post-partum depression). Other examples of clinical group exclusion include children with disabilities or developmental delays, children with specific medical conditions, and specific subgroups of premature infants. While potentially related to relational health, studies that reported on mental health outcomes (depression, anxiety, stress, etc.) but did not also include relational health measures were excluded from this review.

### Screening and Extraction

All records were imported into LitQuest (Fuller-Tyszkiewicz et al., 2025; Grbin et al., 2022) for deduplication and machine learning assisted screening of titles and abstracts. Titles and abstracts were independently double screened against eligibility criteria by four study authors (SV, TEW, GM, GZ) until a stop signal was given by LitQuest. Screening conflicts were resolved by discussion to reach consensus. The full texts of potentially relevant articles were then assessed by two independent study authors against eligibility criteria.

### Changes to Inclusion Criteria During Full Text Screening Phase

One change to inclusion criteria was implemented at the full-text review stage. We initially defined our inclusion criteria to include programs that were delivered to certain population sub-groups (e.g. low-income families, rural families, specific racial groups, pre-term infants) who may be at higher risk of relational problems. The number of articles included after full-text screening was higher than anticipated, with a significant representation of universal studies (i.e., those delivered to all children/families in a region, regardless of risk status). During the data extraction process, we therefore decided to refine the focus of the review to universal studies only. See Table 1 for final inclusion and exclusion criteria for full-text review.

**Table 1:** Inclusion and exclusion criteria for full-text review

Inclusion	Exclusion
Written in English	Not written in English
Human subjects	Non-human subjects
RCT study design	Quasi-experimental, cross-sectional, longitudinal, case-study, review, protocol etc
Program or intervention is delivered universally to all recipients in targeted area, without selection based on assessment of risk Program can be of any length or intensity	Program or intervention is delivered in a clinical setting and/or targeted at specific/indicated population groups based on clinical criteria of relational rupture between infant-parent or other relationships around the child (e.g. foster or adoptive parents, children of parents experiencing domestic violence, substance use, post-partum depression) Program or intervention is delivered to other population sub-groups for example: Children with disabilities or developmental delays (e.g., ASD, cerebral palsy, intellectual disabilities, speech delays) Children with specific medical conditions (e.g., pneumonia, obese/overweight) Premature infants or low birth weight infants Parents with high-risk pregnancies Priority population sub-groups such as low-income, low SES, rural populations, and racial and ethnic groups, including Indigenous^*^
Reports a relational health outcome of interest. These outcomes reflect child exposure to relationships and observations of relationships with and between parents, siblings, grandparents, and caregivers. This may include: Infant/child/foetal attachment, bonding, relationships or interactions with any family member or caregiver (this includes siblings, grandparents etc.) Any form of sensitivity, involvement, interactions, or investment from a form of caregiver Any form of ‘parenting’ that the child may be exposed to, e.g., parenting styles, parenting quality, parenting conflict, feeding practices Any form of parent-parent interactions, e.g., dyadic adjustment, relationship quality or satisfaction, marital conflict etc Any form of family functions, e.g., family conflict, family cohesion, family involvement etc	There is no relational health outcome of interest. This includes: Parental mental health Parental stress Parents’ perceptions of their parenting ability (parenting self-efficacy/ competence/ confidence)
The measurement of the outcome is between pregnancy and 48 months postpartum	The measurement of the outcome is at 4 years of age or older

^*^These studies were originally included. A change to this criterion was implemented at the end of full-text screening resulting in exclusion of these studies from the final article set

Data from articles deemed relevant following full-text screening were extracted using a standardized extraction form. This form included information on sample characteristics, relational health measures, nature of intervention (including control group type), follow-up period, and key findings with respect to relational health outcomes. Following pilot testing of the form, data were extracted by SV, TEW, GZ and GM with 20% cross-checked by TEW and SV.

### Data Collation and Analysis

Relational health outcomes were classified into five types based on the nature of the relationship examined and membership of the relationship. The classification was developed and coded by MF-T in consultation with the other authors. *Intervention setting/location* was post-coded into five major categories and *intervention facilitator* was post-coded into six categories using a classification system agreed by all authors. *Intervention focus* was classified into four categories based on extracted information about intervention details. The classification was developed by TEW in consultation with the other authors.

### Establishing the Living Review

Our intention is to monitor the emergence of new literature in this field following Cochrane and other guidance on living reviews (Elliott et al., 2021; Iannizzi et al., 2023; Synnot, 2019). Periodic updates of this living review will be conducted and available on the SEED Lifespan Systematic Review Hub, a web platform making these and other reviews available for public access. For this, searches will be rerun and output uploaded into LitQuest to allow newly retrieved articles to be screened against the established eligibility criteria. Data will be extracted from included articles using the same methodology described here and synthesised with previous evidence. Timing of publication updates will be based on the impact of the new data on the review conclusions. At each update, we will assess the feasibility and need for continuation of the living mode.

## Results

### Selection of Sources of Evidence

**Fig. 1:**
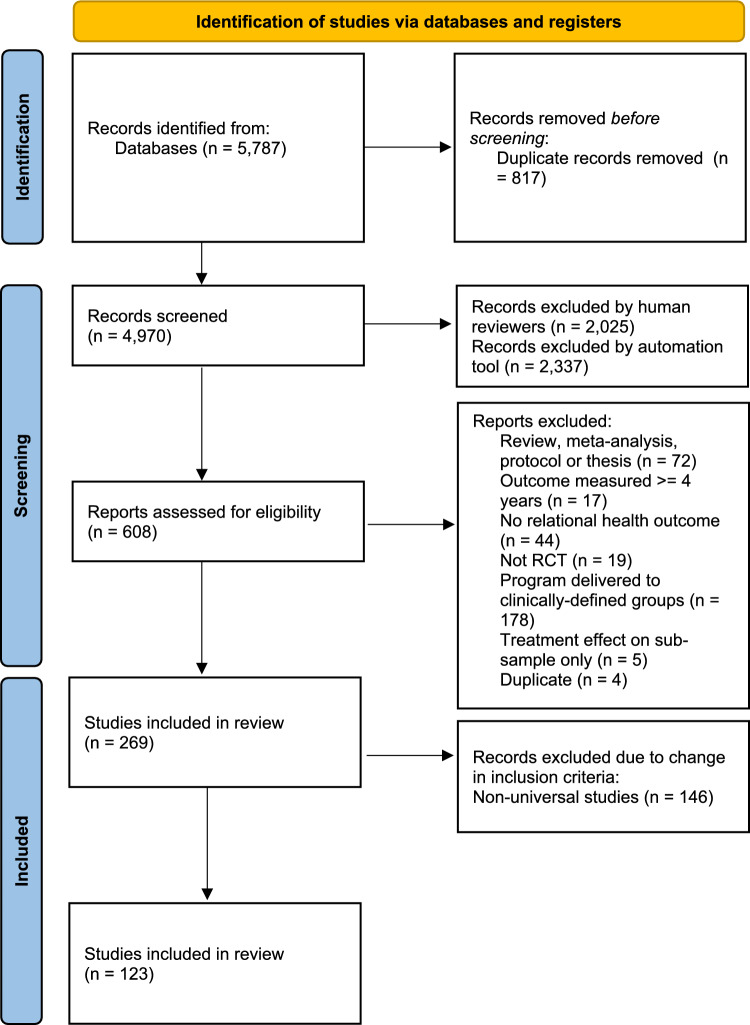
PRISMA flow diagram of the study selection process

The search identified 5787 studies, of which 4970 articles were retained for title and abstract screening following removal of duplicates. At the title and abstract review stage, 2663 (53%) were manually screened, with 2025 excluded. The remaining 2337 articles were excluded by the LitQuest. 608 full texts were accessed and reviewed to determine eligibility for inclusion, with 123 meeting eligibility criteria and thus forming basis for this review. Figure 1 provides the PRISMA flowchart for article retrieval and screening, and details of each included study are provided in Online Resource 2. The 123 retained studies report on 110 unique interventions.

### Study Characteristics

314 relational health-based outcome measures were identified across the 123 retained studies. We broadly categorised these measures as: (i) abuse/neglect, (ii) attachment and bonding, (iii) behavioural support, (iv) emotional support, and (v) relationship quality (see Table 2 for more detailed description).

**Table 2:** Categorising relational health outcomes across included studies

Category	Definition
Abuse/neglect (*n* = 7 effects across 123 studies)	Measures that assessed perceived or actual instances of abuse and/or neglect within the home context Given the severity of these behaviours, these outcomes were separated from other categories rather than treated as extreme negative levels of attachment or relationship quality
Child–parent attachment and parent–child bonding (*n* = 144 effects)	Measures that assessed child-parent attachment, sensitivity of parent to child needs, and bonding behaviours such as skin-to-skin contact These measures were self-rated, partner-rated, or observer-rated
Behavioural support (*n* = 66 effects)	Measures that assessed parent involvement and support for child in a range of behaviours, such as sleep, feeding, and general play While these behavioural supports are likely to foster connection between parent and child, and are likely markers of good relationship quality, measures that focused directly on level and type of support rather than these other relational outcomes were classified as behavioural support
Emotional support (*n* = 14 effects)	Measures that assessed the extent to which parents engaged in behaviours to support emotional needs of their child. This included emotion regulation activities, such as managing instances where their child was emotionally distressed. As with behavioural support, measures were included in this emotional support category rather than other related categories when the measure emphasized the approach to emotional support rather than other relational consequences of this emotional support
Relationship quality (*n* = 87 effects)	This category included perceptions of relationship quality with respect to parent-parent relationships, parent–child relationships, and family relationships (where the survey asked about the family unit as a whole) These measures typically assessed quality and/or satisfaction with these relationships. We also included measures of time availability of parents as a reflection of prioritising time with their child These measures were separated from attachment/bonding and behavioural and emotional supports because the relationship assessments were typically of a global nature, rather than probing specific types of behaviours or specific approaches to parenting (e.g., attachment styles)

Twenty-four studies reported on interventions delivered during the prenatal period (conception to birth), while 65 targeted the postnatal period (birth to three years of age) and 34 targeted both. Fifty-eight of the interventions were entirely or partially delivered in hospital or community health clinics, 59 were delivered at home (with in-person visits, via online resources, or via phone), ten were in other community locations, seven were in research facilities, and 11 did not specify location.

Interventions were facilitated by allied health professionals (*n* = 16), healthcare professionals (*n* = 25), multidisciplinary teams (*n* = 14), researchers (*n* = 26), other background (*n* = 13), students (*n* = 3), or community members (*n* = 3). Ten were self-administered, and background of facilitator was not explicitly stated for 13 studies. All studies focused on one or both parents, except for Moreno (2015), which targeted childcare workers.

In total, there were 74 unique interventions in the retained articles. Of these, 68 were represented by single papers, while 6 had two or more RCTs. These programs with repeated RCTs were Bringing Baby Home (Shapiro, 2011; Shapiro, 2020), Family Foundations (Feinberg et al., 2016; Jones, 2018; Kan & Feinberg, 2015; Solmeyer et al., 2014), INSIGHT (Adams et al., 2018; Harris et al., 2020; Hyczko et al., 2021; Ruggiero, 2021; Savage, 2018), Relational Savouring (Borelli et al., 2023a, 2023b), Brazelton Neonatal Behavioural Assessment (Belsky, 1985; Kristensen et al., 2020; Myers, 1982), and Family Connects (Baziyants et al., 2023; Dodge et al., 2019). In all cases, these interventions focussed primarily within the family microsystem. No intervention addressed wider systemic influences on the functioning of the family microsystem.

These interventions varied in duration and focus. Many focused on enhancing parent knowledge, expectations, and/or skills in parenting, with some of these offering broad range of topic areas (e.g., Dodge et al., 2019; Feinberg et al., 2016) while other studies were focused on specific parenting tasks (e.g., (breast)feeding; Abbass-Dick et al., 2015; Daniels et al., 2014; Harris et al., 2020). Formats were generally fixed, though some studies focused content on needs of the family as assessed in home visits (Dodge et al., 2019). A smaller grouping of studies focused on activities that may foster attachment and bonding, such as singing (e.g., Cevasco et al., 2008; Wulf, 2021a, 2021b), viewing of an ultrasound during pregnancy (Westerneng, 2022; Zachariah Boukydis et al., 2006), memory elicitation to focus on positive experiences with one’s child (Borelli et al., 2023a, 2023b), and infant massage (Suchy et al., 2020).

Table 3 summarises the different intervention foci across retained studies. Half of the studies (55%) reported on interventions focussed on delivering information or skills training to parents. Parent–child interaction was the focus of 36%, 16% on the couple relationship and 14% on providing parents with community and social supports.

**Table 3:** Categorising focus areas of interventions across included studies

Category	Definition
Parent–child interaction (*n* = 45 of 123 studies)	Programs that emphasise activities and strategies to enhance bonding, communication, and positive interactions between parents and their children, before or after birth. They often involve practical activities, such as guided play or responsive parenting techniques, to help parents better understand and respond to their child’s needs
Parental education and skill development (*n* = 66 of 123 studies)	Programs focused on educating parents about child development, parenting skills, and emotional support. It includes educational workshops, informational resources, and coaching
Couple relationship (*n* = 20 of 123 studies)	Programs aimed at strengthening the relationship between parents, addressing communication, conflict resolution, and co-parenting strategies
Parental community and social support (*n* = 17 of 123 studies)	These programs provide parents with access to community resources and peer support networks. They often include group sessions, support groups, and community events that help parents connect with others, share experiences, and receive practical support in parenting

Studies could be classified in more than one category in cases of multiple foci. No studies targeted all four foci, but four studies targeted three of these domains (Abbass-Dick et al., 2015; Kavanagh et al., 2021; Koushede et al., 2017; Wigglesworth et al., 2023). Ten studies were unable to be categorised into these classifications (Baltaci et al., 2023; Çankaya et al., 2024; Kahraman & Gökçe İsbir, 2023; Kawahara et al., 2023; LoCasale-Crouch., 2023; Lukowski et al., 2015; Moreno et al., 2014; Prado et al., 2018; Uncu et al., 2025; Witte et al., 2022)

### Key Findings

Across the 314 outcome measures assessed in these 123 studies, 137 (44%) showed a statistically notable treatment effect (*p* < 0.05). This efficacy varied depending on study characteristics. As detailed in Table 4, parent-parent and mother–child relationships were more common targets of intervention than the father-child relationship, and both also more often exhibited significant improvement in relational health outcomes relative to outcomes from father-child focused interventions. Surprisingly, interventions facilitated by health professionals had lower rates of positive outcomes than interventions facilitated by other groups (e.g., allied health, multidisciplinary teams, researchers, and trained facilitators).

**Table 4:** Proportion of studies with significant improvements in relational outcome by study type

Feature	Levels	Significant intervention effect* (n, %)
Target of intervention	Father–child relationship	12/31 (39%)
	Mother–child relationship	56/121 (46%)
	Parent–parent relationship	64/123 (52%)
Facilitator of intervention	Allied health	27/59 (46%)
	Community member	4/8 (50%)
	Healthcare professional	32/79 (41%)
	Multidisciplinary team	20/35 (57%)
	Not specified	13/24 (54%)
	Other	22/33 (67%)
	Researcher	34/59 (58%)
	Self-guided	7/17 (41%)
	Student	1/3 (33%)
Location of delivery	Home/online/phone	47/108 (44%)
	Hospital/community health clinic	68/144 (47%)
	Other community locations	17/37 (46%)
Outcome measure	Abuse/neglect	1/7 (14%)
	Attachment/bonding	69/144 (48%)
	Behavioural support	31/66 (47%)
	Emotional support	6/14 (43%)
	Relationship quality (parent–child)	9/20 (45%)
	Relationship quality (parent–parent)	33/63 (52%)
	Relationship quality (family unit)	3/4 (75%)
Target period	Prenatal	25/40 (63%)
	Postnatal	87/192 (45%)
	Both	42/82 (51%)

^*^significance was assessed at *p* < .05. Given multiple relational health outcome variables reported in some studies, this table reports on significance per reported outcome (*n* = 314) rather than per study (*n* = 123). In some cases, the levels of a feature do not total 314—this is due to some studies not specifying a given feature

The intervention setting seemed to have little impact on whether there was significant improvement of relational health outcomes. Targeting intervention during the prenatal period had slightly higher rates of significant improvement in relational health outcomes than targeting postnatal period or both prenatal and postnatal periods.

With the exceptions of abuse/neglect (low numbers, low success) and relationship quality for family unit (low numbers), the proportion of studies with significant improvement was reasonably consistent across relational health outcomes. This may in part be due to non-independence of these effects, such that ineffective interventions may exhibit null results across a range of measures, while efficacious studies may positively impact multiple outcomes.

## Discussion

Here we conduct a scoping review to map what evidence-based interventions are available to strengthen the relational ecology of early child development, from families to communities, the connections between them, and the structures that support them. The majority of the 123 interventions focused on the family microsystem, with only 17 interventions classified as focusing on parent community and social support. We found no interventions at broader levels of the social ecology of the early family setting. Within the family, most focused on the mother–child relationship. Few focused on the father-child relationship; however, interventions that focused on parenting could include both. Likewise, few interventions targeting broader kin and non-kin networks were identified. There is a pressing need for more intervention research beyond the microsystem of the immediate family.

### Promising yet Variable Efficacy

Approximately half of the included studies showed significant improvement in the quality of relationships between young children and their parents. A handful of universal programs characterised by multi-component programs with primary focus on promoting early relational health (e.g., Family Foundations; Solmeyer et al., 2014) showed promise with multiple RCTs supporting their efficacy. For the remainder of studies, efficacy was more often demonstrated when the program was: (i) focused on child-mother or parent-parent relationships than sole focus on the child-father relationship; (ii) facilitated by researchers or trained facilitators rather than medical, clinical, or allied health professionals; and (iii) when the program was delivered in the prenatal period rather than postnatal or both prenatal and postnatal periods. Efficacy was also less commonly established for abuse/neglect outcomes, though this is not surprising given the light-touch, universal nature of many included trials in this review.

The lower efficacy for father-child focused interventions is particularly important to understand and may be attributed to bias in who signs up to participate in these interventions. Fathers are difficult to engage in research, and those who participate in these interventions may be more motivated fathers for whom less improvement in relational health may be expected (Magill-Evans et al., 2007; Suchy et al., 2020). Efficacy of these programs may also depend on involvement and connection to partner (Buisman et al., 2023), which was often not a focus of the intervention nor measurement for efficacy estimation. The role of the father may also change across early childhood, with opportunities for building relationships as children develop from dependent infants to more independent toddlers. Providing parenting support and training prior to birth may be particularly beneficial because it provides more time for parents to absorb received information, prepare for birth, and practice taught skills. In contrast, attempts to learn these skills once the child is born may be complicated by initial stress of parenting, loss of sleep, and limited time to commit to this training with these competing demands on their time.

### Opportunities to Expand Targets of Relational Health Programs

Additional focus on the role of fathers and other coparents (particularly in light of limited number and efficacy of extant programs) and other caregivers within the extended family (e.g., grandparents, uncles, and aunts) and extended support systems (e.g., early child care, neighbourhood support groups, family friendly parental workplace) would help to ensure a strong broader network of support around children and their parents within the immediate family setting. Conversely, failure to assess strength of these broader networks miss an opportunity to contextualise evidence of (failed) efficacy of a given program at the level of individual families.

Similarly, this focus of identified universal programs on the more narrowly defined family unit (father, mother, child) also misses opportunities to connect this relational health microsystem with broader structures that may provide further support for parents and child. For instance, to some extent, ability to engage in parenting programs pre- and post-birth are contingent on flexibility in work arrangements, and support from family and friends to prioritise engagement and motivation to continue with these programs. Healthcare providers who are aware of, and actively encourage, their patients to utilise evidence-informed and well received universal services to support the child and family would also help make for a more coherent and comprehensive experience.

### Scope for Intensifying and Scaling Up Programs

Programs such as Baziyants et al. (2023) and Dodge et al. (2019) offered a flexible support program dictated by initial assessment of family needs from scheduled home visits. Similarly, Callejas et al. (2021) tested three levels of care that could potentially be offered according to need. This flexibility in these interventions provides examples of how proportionate universalism (Marmot et al., 2010) may be feasibly applied in the context of supporting relational health of families of newborn children. Importantly, these programs were based on provision of commonly available services (at least within the regions where these study populations resided), suggesting potential for scale-up without need to develop and maintain new services.

Findings from this scoping review should be placed within the context of limitations of this review. Foremost, we have conducted an initial scoping review with the principle aim to describe the lay of the literature across the relational ecology of early childhood development. We did not quantify efficacy of identified studies. Nor did we conduct any risk of bias assessment for any study. This is because studies across the relational ecosystem are currently too heterogenous (diverse measures, varying foci, and non-comparable duration) to allow meaningful analysis and synthesis using conventional systematic and meta-analytic review methods. Our aim from here, however, is to use this scoping review as the foundation for a *living systematic review* when the literature on the relational ecology of early child development becomes sufficiently developed to do so.

Second, our review prioritised identification evidence-based programs at all levels of the relational ecology of early child development. Such a focus ignores arguments for greater uptake of efficacious programs based instead (or as well on) cost-effectiveness and evidence of implementation success. The fact that many trials relied on existing and widely available healthcare services is encouraging, yet the more consistent evidence of efficacy in programs facilitated by people who are not healthcare professionals suggests potential room for improvement in how to integrate existing services into effective relational health care.

A further limitation is exclusion of pre-term infants due to the possibility that universal programs had been revised to be suitable for use in higher risk settings, thus limiting universalism of program. A further review might evaluate the extent to which existing universal programs are suitable for strengthening the relational ecology in ways that would support the health development of preterm infants too (Evans et al., 2014). Finally, while we hope that the present series of reviews spurs interest in relational health, we note that much of the intervention programming in this field is rooted in attachment research traditions, with a focus on adult caregiving behaviour and child attachment behaviour. We found no studies focusing on other relevant aspects of caregiver capacities such as reflective functioning and the quality of emotional interactions. Future research could consider measuring these constructs and intervening to improve the conditions that enable them too.

### Actionable Insights

Current scientific knowledge about what works to enrich the relational ecosystem of early child development is nascent and currently myopically centred on interventions that can be applied at the level of the family microsystem. However, within this specific setting of the broader relational ecology, there are a number of programs, suitable for universal implementation, that have been shown to strengthen the most proximal of all relationships; those between children and their parents within the immediate setting of the family.

Based on the evidence currently available, we make three recommendations for future research:

1. *Considering broader relational ecology* In addition to incorporating more of the family network within these interventions, researchers should also consider the roles of early childhood health and education settings, local community settings, and parents’ workplaces in ensuring relational health. There are also wider opportunities to intervene to create better support services to care for those supporting the health development of the next generation offspring. This includes (but is not limited to) universal health, education, workplace and employment services. Work environments that encourage participation in parenting support programs and provide incentives (whether financial or through onsite programs and/or flexibility of working hours) to participate could provide synergistic inputs into parenting outcomes rather than potentially competing with and undermining these efforts. Finally, there is a need for macrosystemic interventions that give status to family life, that privilege government spending around family life, and that ensures the master narrative of society is pro-family life. These are the elements of a wholistic relational health ecology.
2. *Incorporating other prominent figures in child’s life* More work is needed widening and diversifying the targets of these universal programs. Understandably, many of the identified programs have a primary focus on the mother–child dyad as the primary caring relationship. However, this misses key opportunities to involve other significant adults in the wider care ecology around the young family, including fathers, grandparents, and family friends. Each of these figures may have regular interactions with the child, and these interactions offer opportunities to support the child’s development, and the child’s feeling of security and support. Conversely, where relational styles of these other key figures clash with the way parents attempt to relate to their children, this may have detrimental effects on the child’s overall relational health. Cultural diversity and variability in social networks involved in care should also be considered.
3. *Scaling based on need* Identified programs typically compared against wait-list controls or a treatment as usual comparator. Given variable efficacy across and within studies evidenced across these identified programs, more sophisticated trial designs (e.g., Collins et al., 2007) that allow for personalisation, reallocation, or intensification of program resources may be warranted. In addition to potentially reducing the number of parents who receive ineffective prevention/intervention resources, these trial designs may also enable testing of proportionate universalism-based approaches to deployment of parenting support resources. We also note the absence of implementation trials (Wolfenden et al., 2021) across the programs identified in our scoping review. Such information is vital for identifying enablers and barriers to eventual scale-up of programs found to be efficacious.

Enabling the conditions that support the healthy development of bond between children and their adult carers, form families to communities, the connections between them, and the structures the support them, is central for ensuring a healthy start to life for all children. To do so, however, requires access to high quality interventions at all levels of the relational ecology around the developing child. In this review we show that robust scientific progress has been made in the development of interventions at the level of the family microsystem. However, this is not so for other levels of the relational ecosystem where there are virtually no evidence-based interventions available for universal implementation. This is a significant limitation of the field and a critical barrier to enabling systemic, public health responses, to setting the broader conditions needed to protect and enable quality interactions between children and their adult carers in everyday life. Our continuing inability to drive down rates of vulnerability in early life may, to a large degree, be due to the lack of evidence-based interventions at the outer levels of the relational ecosystem. We recommend that future research pays greater attention to developing effective interventions to address these outer social determinants. To do so would enable a new era of evidence-base public policy capable of strengthening all levels of the relational ecology of early child development.

## Supplementary Information

Below is the link to the electronic supplementary material.

Supplementary file1 (DOCX 91 KB)
