# Supplementary material for: Mapping What Works to Strengthen the Relational Ecology of Early Child Development: A Systematic Scoping Review
Source: Clin Child Fam Psychol Rev. 2026 Apr 17;29(2):241–55. doi: 10.1007/s10567-026-00558-6 (PMC13282253; doi:10.1007/s10567-026-00558-6)
Supplement: Supplementary file 1 — Supplementary file1 (DOCX 91 KB) [file 10567_2026_558_MOESM1_ESM.docx]

**Online Resource 1**

**Search syntax for database search**

| Concept 1 – Outcome  Who/**What**: Child & Family Relational Ecology  Reflects child exposure to relationships and observations of relationships with and between parents, siblings, grandparents, and caregivers | Concept 2 – Outcome  When: First 3 years of life  Reflects the period from conception up to three years post-partum. | Concept 3 –  How |
| --- | --- | --- |
| **[ti,ab]**  (Infan* OR child* OR fetal OR fetus OR foetal OR foetus OR “parent-child” OR “child-parent” OR “parent-infant” or “infant-parent” OR “fetus-parent” OR “parent-fetus” OR “foetus-parent” OR “parent-foetus” OR “mother-child” OR “child-mother” OR “mother-infant” or “infant-mother” OR “fetus- mother” OR “mother-fetus” OR “foetus-mother” OR “parent-child” OR “father-child” OR “child-father” OR “father-infant” or “infant-father” OR “fetus-father” OR “father-fetus” OR “foetus-father” OR “father-foetus” OR  “maternal-child” OR “child-maternal” OR “maternal-infant” or “infant-maternal” OR “fetus-maternal” OR “maternal-fetus” OR “foetus-maternal” OR “maternal-foetus” OR “paternal-child” OR “child-paternal” OR “paternal-infant” or “infant-paternal” OR “fetus-paternal” OR “paternal-fetus” OR “foetus-paternal” OR “paternal-foetus” OR ”parental-child” OR “child-parental” OR “parental-infant” or “infant-parental” OR “fetus-parental” OR “parental-fetus” OR “foetus-parental” OR “parental-foetus”)  **N1** **(attach* OR relations* OR interact* OR bond*)**    OR    (Grandparent* OR grandmother* OR grandfather* OR parent* OR maternal OR paternal OR father* OR mother* OR caregiv* OR alloparent* OR cousin* OR Aunt* OR Uncle* OR Nanny OR Nannies OR “early childcare” OR “early childhood educator” OR friend* OR peer* OR playmate* OR ‘play mate*’ OR ‘class mate* OR classmate*) **N1** (**sensitivity OR involve* OR relation* OR interact* OR investment* OR bond* OR attach* OR availab* OR responsiv* OR warm* OR hostil* OR connect*)**    OR    (Brother OR Sister OR  Sibling) **N1 (Relations* OR conflict* OR interact*)**    OR    **“dyadic synchrony”**  **OR “dyadic attunement”**  **OR “dyadic mutuality”**  **OR Kinship**    OR    **‘Parent-parent’**  **OR** **Interparent***  **OR “parenting”**  **OR “parent* styles”**  **OR “parent* quality”**  **OR “parent* behav*”**  **OR “parent* conflict”**  **OR coparent***  **OR “co-parent*”**    OR    **“Family conflict” OR “Family cohesion”** **OR “Family relations*”** **OR “Family function*”**  **OR “Family interact*”** **OR “Family involve*”**    OR    **“Dyadic adjustment” OR “Relationship quality” OR “relationship conflict” OR “relationship satisfaction” OR** **“Marital quality” OR “marital conflict” OR “Marital satisfaction”**    OR    **“Social network intervention*” OR**  **Egocentr* OR**  **“Whole network*” OR**  **“Network intervention*” OR**  **“Ego network*” OR**  **Egonetwork* OR**  **Sociometr* OR**  **Sociogram* OR**  **Sociomap* OR**  **Sociocentr* OR**  **“Graph theory” OR**  **“Structural network*”** | **[ti,ab]**  Perinatal* OR  Peri-natal  OR Neonatal*  OR Peripartum  OR Puerperium  OR Puerperal*  OR Postnatal*  OR Post-natal  OR Postpart*  OR Childbirth  OR Primipar*  OR “New parent*”  OR “Transition to parent*”  OR “Transition to mother*”  OR “Transition to father*”  OR Baby  OR Infan*  OR Birth  OR Antenatal*  OR Ante-natal  OR Antepartum  OR Pregnan*  OR “Peri-conception”  OR “Periconception”  OR Toddler*  OR “early years”  OR “first year of life”  OR “first 1000 days”    OR (“12 month*” or “12-month*” or “18-month*" or “18 month*” or “two years” or “two-years” OR "three years” OR “three-years” OR “2 years” OR “2-years” OR “3 years” OR “3-years”) N2 (age* OR “life” OR old) | **[ti,ab]**  RCT OR    (Random* N3 (study or studies or trial*)) OR    (Random* N3 (allocat* or assign* or select*)) |

**Online Resource 2**

**Table of study details of included studies**

| **Author (Year) Country**  **Intervention** | **Analytic N** | **Intervention focus, target** | **Intervention target period** | **Intervention location/setting** | **Intervention facilitator** | **Outcome construct (rater)** | **Timing of outcome assessment** | **Significant intervention effects**^2^ |
| --- | --- | --- | --- | --- | --- | --- | --- | --- |
| Abasi et al. (2023), Iran. Couple online training on maternal-fetal attachment | 100 couples | PSESD, CR | Prenatal | Home | PhD student in reproductive health and a psychologist | Maternal-fetal attachment (mother) | 4 weeks post-intervention | Yes – higher maternal-fetal attachment |
| Abbass-Dick et al. (2015), Canada. Coparenting breastfeeding support | 214 couples | PCI, CR | Postnatal | Hospital | NS | Coparenting relationship quality (mother)    Partner support (mother) | 2 days PP, 6- & 12- weeks PP    6- & 12- weeks PP | No |
| Adams et al. (2018), USA. ‘INSIGHT Responsive Parenting Intervention’ | 279 mother-child dyads | PCI | Postnatal | Home, research centre | Research nurses | Frequency of interactive play with child: on the floor, inside/outside (mother) | 8- & 20- weeks PP, 2 years  PP | No |
| Adler-Baeder et al. (2025), USA. ‘Couples Connecting Mindfully’ and ‘ELEVATE’ | 388 parents | PSESD, CR | Postnatal | Community based family resource centres and a single university-based site | Trained professional community educators | Punitive parenting (parent) | Immediate post-program and 6 months follow-up | No |
| Altay et al. (2024), Turkey. Baby calming training | 84 mothers | PSESD | Postnatal | Gynecology and obstetrics services of a public hospital; via phone | Researcher | Maternal attachment (mother) | 4- & 8- weeks PP | Yes – higher maternal attachment (8 weeks PP) |
| Ammerman et al. (2024), USA. ‘Integrated Behavioral Health Prevention’ | 105 mother-child dyads | PSESD | Postnatal | Academic primary care office | Doctoral-level paediatric psychologist | Parenting beliefs and practices: attunement, regularity of routines (mother)  Parenting: building relationships, promoting learning, supporting confidence (researcher) | 6-month follow-up | No  No |
| Baltaci et al. (2023), Turkey. Music interventions | 120 mothers | NC | Prenatal | Hospital – obstetrics outpatient clinics | Researcher | Maternal antenatal attachment (mother) | Post-intervention | Yes – higher maternal antenatal attachment |
| Bayer et al. (2009), Australia. ‘Toddlers Without Tears’ | 589 mothers | PSESD | Postnatal | Maternal and child health (MCH) centre | MCH nurses | Parenting behaviour:  unreasonable expectations, warm nurturing, harsh discipline (mother) | 3 years PP | Yes - lower unreasonable expectations |
| Baziyants et al. (2023), USA. ‘Family Connects’ | 496 families | PCSS | Postnatal | Home | Registered public health nurse | Mother’s parenting behaviour: positive, negative (mother)  Father’s involvement (mother) | 2 years PP | Yes - more positive parenting behaviours  No |
| Belsky et al. (1985), USA. ‘Brazelton Neonatal Behavior Assessment’ | 60 families | PSESD | Postnatal | Hospital | Researcher | Mother-infant interaction (researcher)    Mother-father-infant triad interaction, (researcher) | 1-, 3- & 9 months PP | No  No |
| Binda et al. (2025), Chile. ‘C@nnected’ | 20 mother-child dyads | PCI, PSESD | Postnatal | Online | Trained providers (psychologists) | Maternal bonding (mother) | 2-3 months post-intervention | No |
| Borelli et al. (2023a), USA. ‘Relational Savoring’ | 147 mother-child dyads | PCI | Postnatal | Home | Trained paraprofessionals | Mother’s reflective functioning (mother) | 3 months post- intervention  (~ 2 years PP) | No |
| Borelli et al. (2023b), USA. ‘Relational Savoring’ | 148 mother-child dyads | PCI | Postnatal | Home | Trained paraprofessionals | Closeness to child (mother) | Post-intervention  (~ 20 months PP) | Yes - increased closeness |
| Brown et al. (2000), USA. ‘My Baby U’ | 200 mother-child dyads | PSESD | Both | Home | Self-taught | Mother's responsiveness to infant (paediatrician) | Mid-intervention (~ 6 months PP) | No |
| Buisman et al. (2022), The Netherlands. ‘Prenatal Video-Feedback Intervention to Promote Positive Parenting’ | 73 fathers | PCI | Prenatal | Prenatal health clinics | Researcher | Father ‘s sensitivity (prenatal, via infant simulator) (researcher)  Father’s involvement, (father) | ~ 25 weeks' G &  ~ 9 weeks PP | Yes - increased sensitivity  No |
| Callejas et al. (2021), Spain. ‘Gaining Health and Wellbeing from Birth to Three’ | 87 parents | PSESD | Postnatal | Primary care centres | Healthcare professionals | Attachment bonds with child, feeding (e.g., eating together at mealtimes) & physical activity & play with child (parent)  Satisfaction with family life | Pre-test (mean 10-12 months PP) & post-test (mean 25-27 months PP) | Yes – higher scores on attachment bonds (level 1 group), feeding (level 2 & 3 groups) & physical activity & play with child  (level 2 & 3 groups)  No |
| Çankaya et al. (2024), Turkey. ‘WHO Intrapartum Care Model’ | 128 mothers | NC | Both | Hospital | Researcher midwife | Parental conduct towards infant during initial encounter following birth (observer) | Immediately after birth | Yes – higher number of positive parenting behaviours towards infant |
| Cevasco et al. (2008), USA^1^ Mothers' singing | 54 mother-child dyads | PCI | Postnatal | Hospital, home | Researcher | Mother-infant bonding (mother) | 14-25 days after hospital discharge | No |
| Chang et al. (2015), Taiwan. Music listening | 296 mothers | PCI | Prenatal | Home | NS | Mother's attachment to foetus (mother) | Pre- and post-intervention | No |
| Cheng et al. (2007), Japan. Home visitation | 85 mother-child dyads | PCI | Postnatal | Home | Public health nurse | Mother-infant relationship (researcher) | 10 months PP | Yes - improved relationship (intervention mothers with disturbed relationships at baseline) |
| Christodoulou et al. (2019), South Africa. Home-visiting | 580 mother-child dyads | PSESD | Both | Home | Local women trained to be community health workers | Quality of mother-child interaction (researcher) | 3 years PP | No |
| Chua et al. (2024), Singapore. ‘Parentbot’ | 118 couples | PSESD, PCSS | Both | Online (via app) | Self-guided | Parent-child bonding (parent) | 1- & 3- months PP | No |
| Cirlak et al. (2024), Turkey. Technology-based parent school program | 48 parents | PSESD | Postnatal | Online | Self-guided, researcher | Parenting skills (parent) | Immediately after intervention and 1-month post-intervention | No |
| Coté et al. (2020), USA. 3D ultrasonography plus 3D print | 96 mothers | PCI | Prenatal | Community and university hospital clinics | Clinicians,  ultrasonographers | Mother's attachment to foetus (mother) | 2 weeks post- intervention | Yes - larger increase in attachment |
| Coté et al. (2023), USA. 3D printed models and 3D printed pictures | 184 parents | PCI | Prenatal | University- and clinic- affiliated hospital system | Ultrasonographer | Antenatal attachment (combination of maternal and paternal ratings) | 2 weeks post ultrasound | While interventions did not differ from each other, significant increases in attachment were observed for both |
| Craig et al. (1982), USA. Extra maternal-infant contact | 49 mother-child dyads | PCI | Postnatal | Hospital | Healthcare workers | Mother-infant attachment (mother) | 1 month PP | No |
| Daley-McCoy et al. (2015), England. Low intensity antenatal classes | 46 mothers, 37 fathers | CR | Prenatal | Hospital | Midwives | Couple relationship functioning:  satisfaction, communication (mother, father) | 6 weeks PP | Yes - less deterioration in mother’s relationship satisfaction & father’s ratings of couple communication |
| Daniels et al. (2014), Australia ‘NOURISH’ | 698 mothers | PSESD | Postnatal | Child health clinics | Dietician, psychologist | Parenting: warmth, irritable parenting (mother) | 21 - 27 months PP | No |
| Delaram et al. (2018), Iran. Fetal movement counting | 208 mothers | PCI | Prenatal | NS | Health care providers | Prenatal attachment (mother) | 37 weeks' G | No |
| Dickie et al. (1980), USA. Training in social competence | 38 families | PSESD | Postnatal | NS | Facilitator had expertise in pastoral counselling | Parent contingent responses (researcher)  Rating of overall competence in interaction from home observation  (researcher)  Competence in parent-infant relationship (mother, father)  Other parent’s competence in parent-infant relationship (mother, father) | 8 weeks post- intervention (~ 10 months PP) | Yes - higher parent contingent responses  Yes – differential effects for mothers & fathers and for different forms of interaction (some positive, others negative)  No  Yes – higher ratings of other parent’s competence |
| Dodge et al. (2019), USA. ‘Family Connects’ | 316 families | CR, PCSS | Postnatal | Home | Nurses | Mother’s parenting behaviour: positive, negative (mother)  Father-infant relationship quality (parent NS) | 6 months PP | No  No |
| Dodge et al. (2014), USA. ‘Durham Connects’ | 531 families | PSESD, PCSS | Postnatal | Home | Nurses | Mother’s parenting behaviour: positive, negative (mother)  Father-infant relationship quality (mother)  Partner relationship conflict (mother)  Home environment quality (researcher) | 6 months PP | Yes - more positive maternal parenting behaviours  No  No  Yes - higher home environment quality |
| Dumas et al. (2013), Russia. Skin-to skin contact, rooming-in | 151 mother-child dyads | PCI | Postnatal | Maternity home | NS | Mother-infant interaction during breastfeeding  (researcher) | 4 days PP | Yes- lower affective responsiveness to infant (mothers of swaddled infants who had been separated from infants at birth)  More roughness with infant (mothers separated from infants at birth & mothers with swaddled infants) |
| Ercelik et al. (2023), Turkey. Online infant massage training | 53 mother-child dyads | PCI, PSESD | Postnatal | Online, home | A paediatric nurse with training in infant massage, self-guided | Maternal attachment | 4-weeks (baseline) & 20- weeks PP | Yes – higher maternal attachment (20 weeks PP) |
| Feinberg et al. (2016), USA. ‘Family Foundations’ | 304 couples | PSESD, CR | Both | Health care facilities | Trained facilitators | Coparenting, couple relationship quality, parenting quality (researcher)  Coparenting quality, relationship satisfaction, family violence (parent) | 10 months PP | Yes - more positive coparenting, better couple relationship quality & parenting quality (observed)  Yes - lower relationship satisfaction (unexpected finding) & lower family violence (self-reported) |
| Findik et al. (2025), Turkey. ‘Mindfulness-Based Childbirth and Parenting Program’ | 36 mothers | PSESD | Both | Online | Researcher who had participated in the program | Prenatal attachment (mother) | Baseline and 8-week follow-up | Yes – higher prenatal attachment (8 week follow up) |
| Frankham et al. (2024), Australia. Online childbirth education course | 66 mothers | PSESD | Both | Online | NS | Mother-infant relationship quality | 6-weeks and 6-months PP | No |
| Gambrel et al. (2015), USA^1^ ‘Mindful Transition to Parenthood Program’ | 33 couples | CR, PCSS | Prenatal | Holistic health centres, community spaces, birthing centres | Marriage and family therapist | Relationship satisfaction (parent)  Empathy toward partner (parent)  Perceived empathy of partner toward self (parent) | Pre- and immediately post-intervention | Yes – higher relationship  satisfaction (fathers only)  No  No |
| Georgsson Öhman et al. (2010), Sweden. Ultrasound screening for Down Syndrome | 2026 mothers | PCI | Prenatal | Ultrasound clinics | Midwives, obstetricians | Mother's attachment to foetus (mother) | 24 weeks’ G | Yes – higher attachment |
| Güney et al. (2019), Turkey. Foetal movement counting | 110 mothers | PCI | Prenatal | Family health centres | Researchers | Mother's attachment to foetus (mother) | Pre-test & 4 weeks after training | Yes – higher attachment |
| Halford et al. (2010), Australia. Compared ‘Becoming a Parent (BAP)’ and ‘Couple CARE for Parents (CCP)’ | 71 couples | PSESD, CR | Both | BAP: Home, phone  CCP: Psychology clinic, home | Clinical psychologist | Couple relationship satisfaction (parent)  Self-regulation for effective relationships (parent)  Couple communication (researcher) | ~ 2 months before birth, post-intervention (5 months PP) & 12 months PP | Yes – lower decline in relationship satisfaction in CCP group (women only)  Yes - lower decline in self-regulation in CCP group (women only)  Yes - decrease in negative communication in CCP group, although group differences were attenuated over time |
| Harris et al. (2020), USA. ‘INSIGHT Responsive Parenting Intervention’ | 207 mother-child dyads | PSESD | Postnatal | Home | Nurses | Mother's use of food to soothe (mother) | 18 months PP | Yes – less use of food to soothe |
| Havighurst et al. (2022), Australia. ‘Tuning in to Toddlers (TOTS)’ | 300 parents (89% mothers; 11% fathers) | PSESD | Postnatal | NS | Researchers with program experience and postgraduate qualifications in psychology or education, trained co-facilitators | Empathy (parent) | 12 months post-intervention | Yes - larger increase in empathy |
| Huang et al. (2024), China. Breastfeeding coparenting intervention | 79 couples | PSESD | Both | Researchers (lactation consultant, psychological counsellor, postgraduate student majoring in maternal and child health), community nurses | Hospital, home visits, online (via app) | Coparenting relationship (rater NS) | 1- & 6- months PP | Yes – better coparenting relationships (1- & 6-months PP) |
| Huebner et al. (2000), USA. ‘Dialogic Reading’ | 129 parent-child dyads | PSESD | Postnatal | Public libraries | Children's librarians | Parent’s use of interactive behaviours that characterise dialogic reading (researcher) | Baseline, after sessions 1 and 2, and 3 – 4.5 months post intervention | Yes – increase in frequency of dialogic reading |
| Hyczko et al. (2021), USA. ‘INSIGHT Responsive Parenting Intervention’ | 232 mother-child dyads | PSESD | Postnatal | Home, clinical research centre,  phone | Research nurses | Infant feeding style: pressure (to finish, to soothe, with cereal), restrictive (amount, quality), responsiveness to child cues of satiety (mother)  Controlling feeding practices: pressure to eat, restriction (mother)  Child feeding: monitoring, pressure, restriction (mother) | 28 weeks PP  1-, 2- & 3-years PP  3 years PP | Yes – lower pressure to finish, pressure to soothe & pressure with cereal  Yes - lower pressure to eat  Yes - less restriction by mothers of girls than mothers of boys (intervention group only) |
| Jeong et al. (2024), Tanzania. ‘Engaging Fathers for Effective Child Nutrition and Development in Tanzania’ | 960 households | PSESD | Postnatal | Peer groups (location decided by participants), home visits | Voluntary government community health workers | Parenting: engagement in stimulation, harsh disciplinary practices (mother, father)  Perceptions of the coparenting relationship (mother, father)  Mother-child dyadic interaction (observer)  Father-child dyadic interaction (observer)  Responsive feeding practices (mother) | Baseline (pre-intervention), Midline (5 months after intervention implementation), endline (2 months post intervention) | Yes –improvements in maternal and paternal stimulation (post-intervention).  No differences on harsh disciplinary practices.  Yes – improved perceptions of coparenting relationship (fathers only; post-intervention)  No  No  No |
| Johnston et al. (2006), USA & Canada. Compared ‘Healthy Steps’ with/without ‘PrePare’  component | 343 mothers | PSESD | Both | NS | Trained facilitators with backgrounds in nursing, clinical social work or mental health practice | Nurturing parenting style (mother) | Baseline & 30 months PP | No |
| Jones et al. (2018), USA. ‘Family Foundations’ | 302 couples | PSESD, CR | Both | Health care facilities | Mixed-gender pair of trained group leaders | Co-parenting: positivity, negativity (researcher)  Parenting: positive, negative (researcher)  Triadic relationship quality (researcher)  Negative couple communication | 2 years PP | Yes - lower  co-parenting negativity  Yes - lower negative parenting  Yes - higher triadic relationship quality  Intervention impact was larger for families that had higher levels of negative couple communication at baseline |
| Kahraman et al. (2023), Turkey. Continuous intrapartum supportive care | 59 mothers | NC | Both | Hospital | Research midwife | Maternal attachment (mother) | 6-8 weeks PP | Yes – higher maternal attachment |
| Kan et al. (2015), USA^1^. ‘Family Foundations’ | 167 couples | PSESD, CR | Both | NS | Trained male-female co-leader team | Co-parenting: agreement, parent-based closeness, exposure to conflict, endorsement of partner's parenting, support, undermining (parent)  Parenting behaviour: positivity, negativity, intrusiveness, reactivity to distress (researcher) | 4 – 8 months PP  13 months PP | Yes - mothers’ & fathers’ pre-birth violence perpetration predicted lower father parenting positivity & higher father parenting negativity in control group (effects of pre-birth violence were attenuated in intervention group)  Yes - mothers’ violence perpetration negatively predicted both parents’ reactivity to distress in control group (effects of pre-birth violence were attenuated in intervention group) |
| Kaner et al. (2024), Israel. Online discussion group to support new fathers during the transition to fatherhood | 122 fathers | PSESD, CR | Postnatal | Online | Researcher | Marital quality (father) | NS | Yes – significant decline in marital quality for control group but not intervention group |
| Kavanagh et al. (2021), Australia. Compared ‘Baby Steps Wellbeing’ and ‘Baby Care’ | 248 couples | PSESD, CR, PCSS | Both | Online | Self-guided | Couple relationship satisfaction (mother, father) | Baseline (3^rd^ trimester), 13- & 26- weeks after baseline | Yes – less decline in relationship satisfaction over time in Baby Steps Wellbeing group than Baby Care group |
| Kawahara et al. (2023), Japan. Educational video promoting paternal involvement in childcare | 451 families | NC | Postnatal | Not specified | Self-guided | Paternal involvement in childcare: feeding, diaper change, bathing, changing clothes, putting child to sleep, taking child for walks, playing with child at home (mother) | 3-, 6-, 12- & 18-months PP | Yes – fathers more likely to take their children for walks  No - for all other aspects of childcare |
| Kennedy et al. (2023), England. Brief couple-focused psychotherapeutic intervention | 60 couples | CR | Both | Clinic, home | Highly experienced senior couple psychotherapists | Couple relationship: dyadic adjustment, consensus, satisfaction, cohesion, affectional expression (parent)  Couple conflict tactics (rated for self and partner): negotiation, physical assault, psychological aggression (parent) | 28 weeks G, 6-8 weeks PP, 6 months PP | No |
| Khodakarami et al. (2018), Iran. Life skills counselling | 64 mothers | CR | Prenatal | NS | Researcher with postgraduate qualifications in midwifery counselling | Marital satisfaction (mother) | Pre-intervention, post-intervention, 4 weeks post- intervention | Yes – higher marital satisfaction |
| King et al. (2023), USA. Teaching baby or learning from baby | 66 mother-child dyads | PCI | Postnatal | Laboratory | Researcher | Maternal caregiving behaviour: intrusiveness, sensitivity, cognitive stimulation, positive regard/warmth, negative regard, detachment/  disengagement (researcher) | After delivery of experimental condition instructions | Yes - higher cognitive stimulation and higher intrusiveness among mothers instructed to teach their infant  Lower warmth among mothers instructed to learn from their infant (unexpected finding)  No group differences on sensitivity, negative regard, detachment  /disengagement |
| Koushede et al. (2017), Denmark. Compared antenatal small-class education and auditorium-based lectures | 1,348 mothers | PSESD, CR, PCSS | Both | Local midwifery sites | Midwives,  health visitors | Parenting alliance (mother) | 6 months PP | No |
| Kristensen et al. (2020), Denmark. ‘Newborn Behavioural Observations’ |  | PCI | Postnatal | Home | Health visitors | Mother-infant interaction (mother) | 1-2 weeks, 3 months & 9 months PP | No |
| Leitch et al. (1999), Canada. Infant communication education (based on ‘Keys to Caregiving’) | 29 mother-child dyads | PSESD, CR, PCSS | Prenatal | NS | Self-taught (videotape) | Mother-infant interaction: caregiver, child, contingency, overall scores (researcher) | 24 hrs PP | Yes – higher total score on mother-child interaction &  higher contingency subscale scores for sensitivity to cues & social- emotional growth- fostering behaviour |
| LoCasale-Crouch et al. (2023), USA. Effective Classroom Interactions for Toddler Educators | 34 toddler educators | NC | Postnatal | Online | Instructors | Quality of child-educator interactions: emotional and behavioural support, engaged support for learning  Teaching practices to prevent/reduce challenging behaviours:  emotional guidance, behavioural and engagement guidance, peer interactions, total score | Post-intervention | Yes – higher quality child-educator interactions  (emotional and behavioural support, engaged support for learning)  Yes – higher use of effective teaching practices to prevent/reduce challenging behaviours  (emotional guidance, behavioural and engagement guidance, peer interactions, total score) |
| Lukowski et al. (2015), China. Diaper manipulation | 82 mother-child dyads | NC | Postnatal | Home, study site (NS) | Recruitment agency,  study personnel | Mother- infant interaction: infant’s engagement and positive affect, mother’s engagement and sensitivity, detachment and flat affect, negative regard for child (researcher) | Baseline, 6 weeks after intervention commenced | Yes - higher infant engagement & positive affect, higher maternal engagement & sensitivity |
| Magill-Evans et al. (2007), Canada. Video self-modelling with feedback | 162 father-child dyads | PCI | Postnatal | Home | Trained, experienced home visitors who understood infant development | Parent-child interaction: parent’s sensitivity to cues, response to distress, social-emotional growth-fostering,  cognitive growth- fostering (researcher) | 5- & 8- months PP | Yes - intervention group maintained sensitivity to infant cues, control group decreased in sensitivity. Intervention fathers improved in fostering cognitive growth |
| Matthey et al. (2004), Australia. ‘Preparation for Parenthood’ program with added ‘empathy’ session | 199 couples | PSESD, CR | Both | Hospital | Clinical psychologist and either a female  social worker/  occupational therapist experienced in counselling new parents | Partner awareness (mother, father)  Partner emotional support  (mother, father)  Sharing of infant-care tasks  (mother, father) | 6 weeks PP  Pregnancy, 6 weeks & 6 months PP  6 weeks & 6 months PP | Yes – higher partner awareness (fathers)  No  Yes - more satisfaction with sharing of infant-care tasks at 6 weeks PP (mothers with low self-esteem) |
| Metgud et al. (2023), India. ‘Sit Down and Play’ | 52 parent-child dyads | PCI | Postnatal | Hospital well baby clinic | Sit Down and Play administrators (physical therapists) | Caregiver teaching and play activities (parent)  Verbal interaction between caregiver and child (parent) | 12 months PP | Yes – improvement in parent-child stimulation activities  Yes – improvement in parent-child verbal interactions |
| Midmer et al. (1995), Canada^1^. Prenatal parenting education | 70 couples | PSESD, CR | Prenatal | Hospital | Social workers | Couple relationship: dyadic adjustment, consensus, satisfaction, cohesion, affectional expression (parent) | 2^nd^ trimester, 6 weeks & 6 months PP | Yes- higher dyadic adjustment, consensus (at 6 weeks PP), & cohesion (at 6 months PP) |
| Miller et al. (2025), USA. ‘Smart Beginnings’ (includes ‘PlayReadVIP’ and ‘Family Check Up’) | 327 mother-infant dyads | PCI, PSESD | Postnatal | Well-baby clinic (PlayReadVIP)  Home (Family Check Up) | A non-clinical Bachelor’s-level interventionist (PlayRead VIP)  Master’s level interventionist (Family Check Up) | Cognitive stimulation in the home (mother, observer)  Maternal feeding styles: responsive to satiety, pressuring to finish, indulgent permissive, indulgent soothing (mother) | 6 months PP  24 months PP | Yes – improved cognitive stimulation in the home  Yes – less likely to exhibit a pressuring feeding style  Intervention also had indirect effects on parent feeding styles (higher responsive and lower indulgent styles) via improvements in cognitive stimulation (24 months) |
| Missler et al. (2020), The Netherlands.  Psychoeducational intervention | 138 mothers and 96 partners | PCI, PSESD | Both | Home | Clinical and Developmental psychologist | Parent-infant bonding (parent) | 6 - & 10-weeks PP | No |
| Moreno et al. (2015), USA. ‘Expanding Quality for Infants and Toddlers (EQ)’ | 183 caregivers | NC | Postnatal | Classroom, workplace/outside classroom | EQ instructors/coaches with qualifications in education or human development related fields | Classroom: emotional-behavioural support, support for language and learning (researcher) | Pre-test, post-test (4-6 months later), follow-up (4 months after post-test) | Yes- increased support for learning and language at follow-up (largest in EQ15 group) |
| Murray et al. (2023), UK. Dialogic book-sharing | 218 families | PCI | Postnatal | Children's centres | Trained research facilitators with a background of working in the early years context. | Parent behaviour: sensitivity, cognitive scaffolding, mental state talk (researcher)  Parent-child interaction: general reciprocity, verbal reciprocity (researcher) | Baseline, post-intervention, and 4-6 months post-intervention | Yes – increases for all outcomes post-intervention |
| Murray-Perdue et al. (2024), USA | 202 couples | PSESD, CR | Postnatal | Home | Trained coaches | Maternal and paternal spousal romantic attachment (mother, father)  Maternal and paternal constructive conflict behaviour (researcher) | 6-months PP (pretest) & 12-, 16- & 18 months PP (follow-ups) | Yes- lower maternal attachment security at 18 months PP (unexpected finding; received conflict intervention only)  Yes – higher paternal constructiveness at 18 months PP (received conflict intervention only) |
| Myers et al. (1982), USA^1^. Brazelton intervention | 42 parent-child dyads | PCI | Postnatal | Hospital | Researcher | Mother-infant interactions, father-infant interactions (researcher)  Father’s caretaking (father)  Satisfaction with infant (parent)  Satisfaction /  affection with infant: first felt love, first felt baby was theirs, (parent) | 1 day PP  4 weeks PP  1 day PP  4 weeks PP | No  Yes - higher father involvement in infant caretaking  Yes - higher parent satisfaction with infant at 1 day PP  No |
| Niccols et al. (2008), Canada^1^. ‘Right from the Start’ | 76 mothers | PSESD, PCSS | Postnatal | Convenient locations (NS) | Infant development specialists with backgrounds in psychology, early childhood education, and/or social work. | Infant attachment security (researcher)  Mother’s sensitivity (researcher) | Baseline, post-intervention, 6-month follow-up  Baseline, post-intervention, 6-month follow-up | No  *When intervention attendance was accounted for, attendees in both groups showed higher maternal sensitivity & infant attachment security than non-attendees |
| Niccols et al. (2009), Canada. ‘COPEing with Toddler Behaviour’ | 79 mothers | PSESD, PCSS | Postnatal | Early years centres | Infant development specialists with backgrounds in psychology, early childhood education, and/or social work. | Parenting behaviour: laxness, overactivity (mother)  Parent-child interaction: child-positive, child – negative, mother-positive, dyad – compliance (researcher) | Baseline, post-intervention, 1 month follow-up  Baseline, post-intervention, 1 month follow-up | Yes – lower mother overactivity  Yes - higher positive child behaviour (1 month follow-up), positive mother behaviour (post-test, 1 month follow-up) & dyad compliance (post-test, 1 month follow-up) |
| Oles et al. (2024), Honduras. ‘Proyecto Redes: Con Amor y Cuidados Madres y Bebés Sanos (With Love and Care, Healthy Moms and Babies)’ | 263 parents | PSESD | Postnatal | Home | Community Change Agents (community health workers) | Paternal involvement: father held child, father played with child (parent) | Post-intervention | No |
| Park et al. (2025), South Korea. Mindfulness-based mobile intervention (‘AvecMom’) | 94 mothers | PSESD | Prenatal | Online (via app) | Self-guided | Maternal-fetal attachment: differentiation of self from fetus, interaction with the fetus, attributing characteristics to the fetus, giving of self, maternal role-taking (mother) | Pre-test, post-intervention | Improvements on maternal-fetal attachment: attributing characteristics to fetus &  differentiating self from fetus (post-intervention) |
| Pontoppidan et al. (2016), Denmark. ‘Incredible Years Parents and Babies Program’ | 104 parent-child dyads | PSESD | Postnatal | NS | Trained group leaders | Mother-child interaction (mother) | Baseline, 1-3 weeks  post-intervention | No |
| Popp et al. (2019), Germany. ‘Baby Triple P - Positive Parenting Program’ | 49 parents | PSESD | Both | Face to face (NS), phone | Trained clinical psychologists | Quality of partnership (parent) | Before birth, 10 weeks & 6 months PP | No |
| Prado et al. (2017), Malawi. Maternal supplementation | 669 mothers | NC | Prenatal | Home | Research staff, research nurses | Mother’s caregiving (researcher, mother) | 6 months PP | No |
| Reilly (2025), USA. Loving-Kindness Meditation | 136 parents | PSESD | Postnatal | Online | Researcher | Parent sensitivity (researcher) | Post-intervention | No |
| Rheinheimer et al. (2022), The Netherlands. Skin-to-skin contact | 116 mother-child dyads | PCI | Both | Home | Researcher | Quality of mother’s caregiving (researcher)  Mother-infant adrenocortical synchrony (cortisol samples) | 5 weeks PP | No |
| Robertson et al. (2018), USA. Contingent lullaby music | 45 mother-child dyads | PCI | Postnatal | Medical centres, birthing centre | Researcher | Parent-infant interaction; mother’s responsiveness, warmth (researcher)  Frequency of interaction behaviour: touching, looking, talking, smiling, infant response, and singing  (researcher) | 6 weeks PP | Yes – higher mother responsiveness & warmth  Yes - more frequent mother-infant eye contact, mother-infant talking, infant response, & singing |
| Ruggiero et al. (2021), USA. ‘INSIGHT Responsive Parenting Intervention’ | 232 mother-child dyads | PSESD | Postnatal | Home | Research nurses | Parent feeding practices: restriction, pressure, food to soothe, food as a reward (mother) | 3 years PP | Yes - less pressure to eat, food to soothe, & food as a reward |
| Sabancı Baransel (2023), Turkey. Attachment-based intervention program | 154 mothers |  | Prenatal | Hospital outpatient clinics | Researchers | Maternal antenatal attachment: quality of attachment, time spent on attachment, total score (mother) | Pre- & post-test (days 7 later) | Yes – increased quality of attachment, time spent on attachment, and overall attachment |
| Sandborg et al. (2025), Australia. ‘Let’s Grow’ app | 1165 parents | PSESD, PCSS | Postnatal | Online (via app) | Self-guided | Parental behaviours – coparticipation (parent)  Parental modelling (parent)  Parental behaviours: family rules, family routines (parent) | Baseline, 6-month follow-up | No |
| Santelices et al. (2010), Chile. ‘Promoting Secure Attachment’ | 72 mother-child dyads | PCI | Both | Health centres | Researchers | Infant attachment (researcher) | Post intervention (during first year PP) | No |
| Sari Ozturk et al. (2023), Turkey. Mandala activity and technology-based breastfeeding program | 66 mother-child dyads | PCI | Both | Hospital | Researchers | Mother-infant attachment (mother) | 1 week & 2 months PP | Yes – higher attachment |
| Savage et al. (2018), USA. ‘INSIGHT Responsive Parenting Intervention’ | 279 mother-child dyads | PSESD | Postnatal | Home | Research nurses | Parent feeding practices; feeding to soothe - emotional, contextual (mother)  Infant feeding style: pressure (to finish, to soothe, with cereal), restrictive (amount, diet quality) responsive satiety (mother) | 8-,16-, 32- and 44-weeks PP  28 weeks PP | Yes – less frequent use of feeding to soothe in response to emotional or contextual factors  Yes - lower pressure to finish & use of feeding to soothe |
| Schroeder et al. (2015), USA. ‘Growing Leaps and Bounds’ | 232 families | PSESD | Postnatal | Health centres | Paediatricians, nurse practitioners, clinic staff | Child feeding practices – dietary restriction, pressure to eat, and monitoring (parent) | 12- & 24-months PP | Yes – more dietary restriction & more active in monitoring child feeding (both at 24 months) |
| Selkirk et al. (2006), Australia. Midwife-led postnatal debriefing | 143 mothers | PSESD | Postnatal | Hospital | Hospital midwives | Couple dyadic adjustment (mother) | 3^rd^ trimester, 3 months PP | No |
| Setodeh et al. (2018), Iran. Paternal-attachment training skills | 150 couples | PCI, PSESD | Prenatal | NS | Researchers | Mother-neonatal attachment (researcher) | 24 hours PP | Yes – higher attachment |
| Shapiro et al. (2011), USA. ‘Bringing Baby Home’ | 181 couples | PSESD, CR | Both | Hospital | Birth preparation teachers | Mother-father-infant triadic interaction: mother engaged and competitive, father engaged and competitive, calm and complementary play, parents coordinated and baby disengaged (researcher) | 3 months PP | Yes – rated lower on ‘father engaged & competitive’, & ‘mother engaged & competitive’ |
| Shapiro et al. (2020), USA. ‘Bringing Baby Home’ | 119 couples | PSESD, CR | Both | Hospital | Birth preparation teachers | Father’s involvement in parenting tasks (parent) | 3 months PP | Yes – more equal distribution of parenting tasks |
| Shariat et al. (2017), Iran. Psychological intervention | 71 mothers | PSESD | Prenatal | Maternity clinics | Clinical psychologist | Mother-infant attachment, (mother) | Baseline, 1-, 3- & 7- days PP, 1-, 3-, 6-, 8 & 12- months PP | Yes – larger increase in attachment |
| Shen et al. (2021), Taiwan. Non-supervised aerobic exercise intervention | 123 mothers | PCI | Prenatal | Home | DVD of a prenatal yoga teacher | Mother’s attachment to foetus (mother) | 1- & 3-months post intervention | Yes – higher attachment |
| Shorey et al. (2023), Singapore. ‘Supportive Parenting App’ | 200 couples | PCSS | Both | Hospital, home | Mobile app, peer volunteers | Parent-infant bonding (mother) | Baseline, 1-, 2, - 4-, 6-, 9- & 12 months PP | Yes - higher parent-infant bonding in control group at 6 months PP (unexpected finding) |
| Shorey et al. (2017), Singapore. ‘Home-but not Alone’ | 125 couples | PSESD, PCSS | Postnatal | Home | Research assistant, mobile app, clinical staff, peers | Perceived social support from partner (parent) | Baseline, 4 weeks PP | Yes – higher social support from partner |
| Shorey et al. (2019), Singapore. ‘Supportive Educational Parenting Program’ | 118 couples | PCSS | Both | Hospital | Research assistant, mobile app | Parent-infant bonding (mother) | Baseline, immediate PP, 1- & 3 months PP | Yes – higher parent-infant bonding (1-&3-months PP) |
| Smith et al. (2023), USA. ‘Music Together’ | 75 parent-child dyads | PCI | Postnatal | Local music studio | Researcher | Mother-child relationship: mother’s warmth/sensitivity, negative affect/harshness, intrusiveness (researcher) | Baseline, 6, 12, 24 months post-intervention | Decreased mother harshness (from baseline to 12 months & 6 months to 12 months) & mother intrusiveness (between 6-12 months) |
| Solmeyer et al. (2014), USA. ‘Family Foundations’ | 132 couples | PSESD | Both | Health care facilities | Trained male-female co-leader team | Coparenting positivity: warmth and cooperation (researcher)  Child adjustment problems | 1 year PP  3 years PP | No  Yes – competition in coparenting mediated intervention effects on child adjustment problems for fathers (for sons & daughters) & mothers (for sons only) |
| Sönmez et al. (2025), Turkey. Hybrid breastfeeding counselling | 90 mothers | PSESD | Both | Hospital, online | Researcher | Mother-infant bonding | 1-5 days PP (pre-test) & 13-24 weeks PP (post-test) | Yes - improved mother-infant bonding (post-test) |
| Sosa et al. (1980), Guatemala. Supportive birth companion | 40 mothers | PCSS | Prenatal | Hospital | Trained support companions | Mother-infant interaction: handling of infant, any verbal or nonverbal communication with infant & nursing behaviours (researcher) | Immediately PP | Yes – more frequent stroking and talking to infants |
| Suchy et al. (2020), USA. Infant massage instruction | 98 fathers | PCI | Postnatal | Hospital, community health centre | Nurses, certified massage instructor | Father-infant interaction: verbal interaction, smiling, eye contact,  fingertip touching, whole-hand touching, total score (researcher) | 12 – 48 hours PP | Yes – more total interactions & fingertip touching in fathers observed immediately after watching video, but less verbal interaction (unexpected finding) in this group |
| Taylor et al. (1985), USA. Extra physical contact immediately after birth | 78 mothers- child dyads | PCI | Postnatal | Hospital, home | Nursing staff | Mother-infant interaction (researcher) | 2 days & 1 month PP | No |
| Toney et al. (1983), USA. Father holding newborn post-delivery | 37 father-child dyads | PCI | Postnatal | Hospital | Graduate nursing students | Father-infant interaction: verbal interaction, smiling, eye contact,  fingertip touching, whole-hand touching, (researcher) | 12 – 36 hours PP | No |
| Turk Dudukcu et al. (2022), Turkey. Health promotion program | 64 mother-child dyads | PSESD | Both | Family health centre | Researchers | Mother’s attachment (mother) | 36 weeks G, 8 - & 24weeks PP | Yes - higher mother’s attachment (8- & 24- weeks PP) |
| Uçakcı et al. (2023), Turkey. Online health training/counselling and ‘Progressive Muscle Relaxation Exercise (PMRE)’ | 52 mothers | PSESD | Both | Home via videocall | Researcher | Maternal attachment | 6 weeks PP | Yes – higher maternal attachment |
| Uncu et al. (2025a), Turkey. Father birth attendance | 103 fathers | NC | Both | Hospital | Researcher | Paternal attachment (father) | 2 weeks PP | Yes – higher paternal attachment |
| Uncu et al. (2025b), Turkey. Newborn massage training | 54 mothers | PCI | Both | Online | Researcher | Maternal attachment (mother) | 6- &10-weeks PP | Yes – higher maternal attachment |
| van Vliet et al. (2022), The Netherlands. ‘Video intervention to promote positive parenting–feeding infants’ | 246 mother-child dyads | PCI | Postnatal | Home | Researchers | Mother’s responsiveness to satiety cues  (researcher)  Mother’s sensitivity (researcher)  Mother’s pressure to eat  (researcher)  Mother’s responsive feeding (mother)  Mother’s pressure to eat (mother) | Baseline, 18 months PP, 24 months PP  Baseline, 18 months PP, 24 months PP  18 months PP, 24 months PP  18 months PP, 24 months PP  18 months PP, 24 months PP | Yes–higher responsivity to satiety cues in VIPP and COMBI groups than AC group (18 months PP)  No  No  Yes - higher responsive feeding in COMBI group than RVE & AC groups (18 months PP)  Yes - less pressure to eat in VIPP & COMBI groups than RVE group (18 months PP) |
| Verhees et al. (2023), The Netherlands. Baby carrier for fathers | 80 fathers | PCI | Postnatal | Home | Researcher | Paternal sensitivity (researcher)  Paternal involvement (father) | Baseline and post-intervention | No |
| Vlasblom et al. (2020), The Netherlands. ‘BBOFT+’ program | 1,195 parent-child dyads | PSESD | Postnatal | Well-baby clinics | Community physicians, nurses | Parenting style: parental warmth, control (parent)  Parenting practices relating to eating and activity - limit setting, control, monitoring, discipline, reinforcement (parent) | 14- & 36- months PP (warmth), 36 months PP (control)    36 months PP | Yes – higher parental control |
| Waldenström et al. (1999), Sweden Birth centre care | 1143 fathers | PSESD | Both | Hospital | Midwives | Father involvement in birth process (father)  Impact of birth attendance on partner relationship (father)  Impact of birth attendance on immediate feelings for newborn (father)  Father home from work within 2 weeks of birth, number of days home (father)  Father taking/already planned additional parental leave, total planned parental leave (months)  Changed diapers during the last 24h, number of diapers changed (father)  Bathed or washed baby during the last 24h (father)  Total time (in hours) spent with baby during the last 24h (father) | 2 months PP | No  No  No  Yes – father spent more days at home during first two weeks  No  No  No  No |
| Walter et al. (2019), Germany. ‘SAFE – Secure Attachment Family Education’ | 104 mothers, 77 fathers | PSESD, PCSS | Both | Hospital | Trained health and social-service professionals | Infant-father attachment (researcher)  Infant-mother attachment (researcher) | 18 months PP | Yes – higher % of infants were securely attached to their fathers  No |
| Weiland et al. (2023), USA. ‘Nurture®’ program | 201 mothers | PSESD, PCSS | Both | Researcher and mentors (mostly teachers, nurses or social workers) | Home | Paternal involvement (mother) | 4-& 18-months follow-up | No |
| Westerneng et al. (2022), The Netherlands. Third trimester routine ultrasound | 1,275 mothers | PCI | Prenatal | Primary care midwifery practices, ultrasound centres, hospitals | Sonographers | Mother’s bonding to foetus (mother) | between 20- & 27-weeks' G, 32 weeks' G | Yes- higher bonding (intervention mothers with low bonding at baseline) |
| Wigglesworth et al. (2023), England. Sling provision and training | 55 mother-child dyads | PCI, PSESD, PCSS | Postnatal | Peer volunteers and trained staff | Sling library | Mother-infant relationship | 0-6 weeks PP (baseline), 12 weeks post-baseline | No |
| Witte et al. (2022), The Netherlands. Intranasal administration of oxytocin and vasopressin | 70 father-child dyads | NC | Postnatal | NS | Researchers | Father’s sensitivity (researcher)  Challenging parenting behaviours (with and without toys) (researcher) | After hormone administration (2-12 months PP) | Yes - lower paternal sensitivity with oxytocin (unexpected finding)  No |
| Witte et al. (2024), The Netherlands. Baby carrier for fathers | 80 fathers | PCI | Postnatal | Researcher | Home | Language and early literacy practices (father) | Baseline, post-intervention, & 5 months post-intervention | No |
| Wulff et al. (2021a), Germany. Prenatal music intervention | 172 mothers | PCI | Prenatal | Hospital, home | Music therapist | Mother’s antenatal attachment (mother)  Perceived closeness to infant (mother) | Pre-intervention, post-intervention (36th weeks G) | No  Yes – largest increase in perceived closeness with infant in singing group |
| Wulff et al. (2021b), Germany. Mother-infant singing intervention | 120 mother-child dyads | PCI | Postnatal | Hospital, home | Music therapist | Mother-infant attachment (mother)  Perceived closeness to infant(mother) | 48 hours PP (baseline), 2 weeks after baseline (T1), immediately pre- and post- intervention, 12 weeks PP (T2) | No  Yes – increase in perceived closeness to infant between pre- and post- intervention |
| Yildirim et al. (2023), Turkey. ‘Kangaroo Care’ | 90 fathers | PCI | Postnatal | Researcher | Hospital | Father-infant attachment (father) | 3 months PP | Yes – higher father-infant attachment |
| Yildirim et al. (2025), Turkey. Infant care training for new fathers | 63 fathers | PSESD | Postnatal | Hospital, phone call | NS | Father-infant attachment (father)  Baby care participation (father) | 3 months PP  2-, 3-, 4-, 8- & 12 weeks PP | Yes- higher father-infant attachment  Yes – higher participation in baby care |
| Zachariah Boukydis et al. (2006), USA. Prenatal ultrasound consultation | 52 mothers | PCI | Prenatal | Hospital | Sonographer | Mother’s attachment to foetus: overall score and attributing characteristics to foetus (mother) | Immediately before and after ultrasound examination | Yes- increase in both outcomes |

Notes: PCI = Parent-child interaction. CR = Couple relationship. PSESD = Parental support, education and skill development.

PCSS = Parental community and social support. NC = Not classified. NS=Not specified. PP= Post partum. G = Gestation. ^1^based on author’s affiliation. ^2^Sig group differences are with respect to the control group and in expected direction unless otherwise stated. Statistical significance threshold is p<0.05.

**Online Resource 2**

**Table of study details of included studies**

| **Author (Year) Country**  **Intervention** | **Analytic N** | **Intervention focus, target** | **Intervention target period** | **Intervention location/setting** | **Intervention facilitator** | **Outcome construct (rater)** | **Timing of outcome assessment** | **Significant intervention effects**^2^ |
| --- | --- | --- | --- | --- | --- | --- | --- | --- |
| Abasi et al. (2023), Iran. Couple online training on maternal-fetal attachment | 100 couples | PSESD, CR | Prenatal | Home | PhD student in reproductive health and a psychologist | Maternal-fetal attachment (mother) | 4 weeks post-intervention | Yes – higher maternal-fetal attachment |
| Abbass-Dick et al. (2015), Canada. Coparenting breastfeeding support | 214 couples | PCI, CR | Postnatal | Hospital | NS | Coparenting relationship quality (mother)    Partner support (mother) | 2 days PP, 6- & 12- weeks PP    6- & 12- weeks PP | No |
| Adams et al. (2018), USA. ‘INSIGHT Responsive Parenting Intervention’ | 279 mother-child dyads | PCI | Postnatal | Home, research centre | Research nurses | Frequency of interactive play with child: on the floor, inside/outside (mother) | 8- & 20- weeks PP, 2 years  PP | No |
| Adler-Baeder et al. (2025), USA. ‘Couples Connecting Mindfully’ and ‘ELEVATE’ | 388 parents | PSESD, CR | Postnatal | Community based family resource centres and a single university-based site | Trained professional community educators | Punitive parenting (parent) | Immediate post-program and 6 months follow-up | No |
| Altay et al. (2024), Turkey. Baby calming training | 84 mothers | PSESD | Postnatal | Gynecology and obstetrics services of a public hospital; via phone | Researcher | Maternal attachment (mother) | 4- & 8- weeks PP | Yes – higher maternal attachment (8 weeks PP) |
| Ammerman et al. (2024), USA. ‘Integrated Behavioral Health Prevention’ | 105 mother-child dyads | PSESD | Postnatal | Academic primary care office | Doctoral-level paediatric psychologist | Parenting beliefs and practices: attunement, regularity of routines (mother)  Parenting: building relationships, promoting learning, supporting confidence (researcher) | 6-month follow-up | No  No |
| Baltaci et al. (2023), Turkey. Music interventions | 120 mothers | NC | Prenatal | Hospital – obstetrics outpatient clinics | Researcher | Maternal antenatal attachment (mother) | Post-intervention | Yes – higher maternal antenatal attachment |
| Bayer et al. (2009), Australia. ‘Toddlers Without Tears’ | 589 mothers | PSESD | Postnatal | Maternal and child health (MCH) centre | MCH nurses | Parenting behaviour:  unreasonable expectations, warm nurturing, harsh discipline (mother) | 3 years PP | Yes - lower unreasonable expectations |
| Baziyants et al. (2023), USA. ‘Family Connects’ | 496 families | PCSS | Postnatal | Home | Registered public health nurse | Mother’s parenting behaviour: positive, negative (mother)  Father’s involvement (mother) | 2 years PP | Yes - more positive parenting behaviours  No |
| Belsky et al. (1985), USA. ‘Brazelton Neonatal Behavior Assessment’ | 60 families | PSESD | Postnatal | Hospital | Researcher | Mother-infant interaction (researcher)    Mother-father-infant triad interaction, (researcher) | 1-, 3- & 9 months PP | No  No |
| Binda et al. (2025), Chile. ‘C@nnected’ | 20 mother-child dyads | PCI, PSESD | Postnatal | Online | Trained providers (psychologists) | Maternal bonding (mother) | 2-3 months post-intervention | No |
| Borelli et al. (2023a), USA. ‘Relational Savoring’ | 147 mother-child dyads | PCI | Postnatal | Home | Trained paraprofessionals | Mother’s reflective functioning (mother) | 3 months post- intervention  (~ 2 years PP) | No |
| Borelli et al. (2023b), USA. ‘Relational Savoring’ | 148 mother-child dyads | PCI | Postnatal | Home | Trained paraprofessionals | Closeness to child (mother) | Post-intervention  (~ 20 months PP) | Yes - increased closeness |
| Brown et al. (2000), USA. ‘My Baby U’ | 200 mother-child dyads | PSESD | Both | Home | Self-taught | Mother's responsiveness to infant (paediatrician) | Mid-intervention (~ 6 months PP) | No |
| Buisman et al. (2022), The Netherlands. ‘Prenatal Video-Feedback Intervention to Promote Positive Parenting’ | 73 fathers | PCI | Prenatal | Prenatal health clinics | Researcher | Father ‘s sensitivity (prenatal, via infant simulator) (researcher)  Father’s involvement, (father) | ~ 25 weeks' G &  ~ 9 weeks PP | Yes - increased sensitivity  No |
| Callejas et al. (2021), Spain. ‘Gaining Health and Wellbeing from Birth to Three’ | 87 parents | PSESD | Postnatal | Primary care centres | Healthcare professionals | Attachment bonds with child, feeding (e.g., eating together at mealtimes) & physical activity & play with child (parent)  Satisfaction with family life | Pre-test (mean 10-12 months PP) & post-test (mean 25-27 months PP) | Yes – higher scores on attachment bonds (level 1 group), feeding (level 2 & 3 groups) & physical activity & play with child  (level 2 & 3 groups)  No |
| Çankaya et al. (2024), Turkey. ‘WHO Intrapartum Care Model’ | 128 mothers | NC | Both | Hospital | Researcher midwife | Parental conduct towards infant during initial encounter following birth (observer) | Immediately after birth | Yes – higher number of positive parenting behaviours towards infant |
| Cevasco et al. (2008), USA^1^ Mothers' singing | 54 mother-child dyads | PCI | Postnatal | Hospital, home | Researcher | Mother-infant bonding (mother) | 14-25 days after hospital discharge | No |
| Chang et al. (2015), Taiwan. Music listening | 296 mothers | PCI | Prenatal | Home | NS | Mother's attachment to foetus (mother) | Pre- and post-intervention | No |
| Cheng et al. (2007), Japan. Home visitation | 85 mother-child dyads | PCI | Postnatal | Home | Public health nurse | Mother-infant relationship (researcher) | 10 months PP | Yes - improved relationship (intervention mothers with disturbed relationships at baseline) |
| Christodoulou et al. (2019), South Africa. Home-visiting | 580 mother-child dyads | PSESD | Both | Home | Local women trained to be community health workers | Quality of mother-child interaction (researcher) | 3 years PP | No |
| Chua et al. (2024), Singapore. ‘Parentbot’ | 118 couples | PSESD, PCSS | Both | Online (via app) | Self-guided | Parent-child bonding (parent) | 1- & 3- months PP | No |
| Cirlak et al. (2024), Turkey. Technology-based parent school program | 48 parents | PSESD | Postnatal | Online | Self-guided, researcher | Parenting skills (parent) | Immediately after intervention and 1-month post-intervention | No |
| Coté et al. (2020), USA. 3D ultrasonography plus 3D print | 96 mothers | PCI | Prenatal | Community and university hospital clinics | Clinicians,  ultrasonographers | Mother's attachment to foetus (mother) | 2 weeks post- intervention | Yes - larger increase in attachment |
| Coté et al. (2023), USA. 3D printed models and 3D printed pictures | 184 parents | PCI | Prenatal | University- and clinic- affiliated hospital system | Ultrasonographer | Antenatal attachment (combination of maternal and paternal ratings) | 2 weeks post ultrasound | While interventions did not differ from each other, significant increases in attachment were observed for both |
| Craig et al. (1982), USA. Extra maternal-infant contact | 49 mother-child dyads | PCI | Postnatal | Hospital | Healthcare workers | Mother-infant attachment (mother) | 1 month PP | No |
| Daley-McCoy et al. (2015), England. Low intensity antenatal classes | 46 mothers, 37 fathers | CR | Prenatal | Hospital | Midwives | Couple relationship functioning:  satisfaction, communication (mother, father) | 6 weeks PP | Yes - less deterioration in mother’s relationship satisfaction & father’s ratings of couple communication |
| Daniels et al. (2014), Australia ‘NOURISH’ | 698 mothers | PSESD | Postnatal | Child health clinics | Dietician, psychologist | Parenting: warmth, irritable parenting (mother) | 21 - 27 months PP | No |
| Delaram et al. (2018), Iran. Fetal movement counting | 208 mothers | PCI | Prenatal | NS | Health care providers | Prenatal attachment (mother) | 37 weeks' G | No |
| Dickie et al. (1980), USA. Training in social competence | 38 families | PSESD | Postnatal | NS | Facilitator had expertise in pastoral counselling | Parent contingent responses (researcher)  Rating of overall competence in interaction from home observation  (researcher)  Competence in parent-infant relationship (mother, father)  Other parent’s competence in parent-infant relationship (mother, father) | 8 weeks post- intervention (~ 10 months PP) | Yes - higher parent contingent responses  Yes – differential effects for mothers & fathers and for different forms of interaction (some positive, others negative)  No  Yes – higher ratings of other parent’s competence |
| Dodge et al. (2019), USA. ‘Family Connects’ | 316 families | CR, PCSS | Postnatal | Home | Nurses | Mother’s parenting behaviour: positive, negative (mother)  Father-infant relationship quality (parent NS) | 6 months PP | No  No |
| Dodge et al. (2014), USA. ‘Durham Connects’ | 531 families | PSESD, PCSS | Postnatal | Home | Nurses | Mother’s parenting behaviour: positive, negative (mother)  Father-infant relationship quality (mother)  Partner relationship conflict (mother)  Home environment quality (researcher) | 6 months PP | Yes - more positive maternal parenting behaviours  No  No  Yes - higher home environment quality |
| Dumas et al. (2013), Russia. Skin-to skin contact, rooming-in | 151 mother-child dyads | PCI | Postnatal | Maternity home | NS | Mother-infant interaction during breastfeeding  (researcher) | 4 days PP | Yes- lower affective responsiveness to infant (mothers of swaddled infants who had been separated from infants at birth)  More roughness with infant (mothers separated from infants at birth & mothers with swaddled infants) |
| Ercelik et al. (2023), Turkey. Online infant massage training | 53 mother-child dyads | PCI, PSESD | Postnatal | Online, home | A paediatric nurse with training in infant massage, self-guided | Maternal attachment | 4-weeks (baseline) & 20- weeks PP | Yes – higher maternal attachment (20 weeks PP) |
| Feinberg et al. (2016), USA. ‘Family Foundations’ | 304 couples | PSESD, CR | Both | Health care facilities | Trained facilitators | Coparenting, couple relationship quality, parenting quality (researcher)  Coparenting quality, relationship satisfaction, family violence (parent) | 10 months PP | Yes - more positive coparenting, better couple relationship quality & parenting quality (observed)  Yes - lower relationship satisfaction (unexpected finding) & lower family violence (self-reported) |
| Findik et al. (2025), Turkey. ‘Mindfulness-Based Childbirth and Parenting Program’ | 36 mothers | PSESD | Both | Online | Researcher who had participated in the program | Prenatal attachment (mother) | Baseline and 8-week follow-up | Yes – higher prenatal attachment (8 week follow up) |
| Frankham et al. (2024), Australia. Online childbirth education course | 66 mothers | PSESD | Both | Online | NS | Mother-infant relationship quality | 6-weeks and 6-months PP | No |
| Gambrel et al. (2015), USA^1^ ‘Mindful Transition to Parenthood Program’ | 33 couples | CR, PCSS | Prenatal | Holistic health centres, community spaces, birthing centres | Marriage and family therapist | Relationship satisfaction (parent)  Empathy toward partner (parent)  Perceived empathy of partner toward self (parent) | Pre- and immediately post-intervention | Yes – higher relationship  satisfaction (fathers only)  No  No |
| Georgsson Öhman et al. (2010), Sweden. Ultrasound screening for Down Syndrome | 2026 mothers | PCI | Prenatal | Ultrasound clinics | Midwives, obstetricians | Mother's attachment to foetus (mother) | 24 weeks’ G | Yes – higher attachment |
| Güney et al. (2019), Turkey. Foetal movement counting | 110 mothers | PCI | Prenatal | Family health centres | Researchers | Mother's attachment to foetus (mother) | Pre-test & 4 weeks after training | Yes – higher attachment |
| Halford et al. (2010), Australia. Compared ‘Becoming a Parent (BAP)’ and ‘Couple CARE for Parents (CCP)’ | 71 couples | PSESD, CR | Both | BAP: Home, phone  CCP: Psychology clinic, home | Clinical psychologist | Couple relationship satisfaction (parent)  Self-regulation for effective relationships (parent)  Couple communication (researcher) | ~ 2 months before birth, post-intervention (5 months PP) & 12 months PP | Yes – lower decline in relationship satisfaction in CCP group (women only)  Yes - lower decline in self-regulation in CCP group (women only)  Yes - decrease in negative communication in CCP group, although group differences were attenuated over time |
| Harris et al. (2020), USA. ‘INSIGHT Responsive Parenting Intervention’ | 207 mother-child dyads | PSESD | Postnatal | Home | Nurses | Mother's use of food to soothe (mother) | 18 months PP | Yes – less use of food to soothe |
| Havighurst et al. (2022), Australia. ‘Tuning in to Toddlers (TOTS)’ | 300 parents (89% mothers; 11% fathers) | PSESD | Postnatal | NS | Researchers with program experience and postgraduate qualifications in psychology or education, trained co-facilitators | Empathy (parent) | 12 months post-intervention | Yes - larger increase in empathy |
| Huang et al. (2024), China. Breastfeeding coparenting intervention | 79 couples | PSESD | Both | Researchers (lactation consultant, psychological counsellor, postgraduate student majoring in maternal and child health), community nurses | Hospital, home visits, online (via app) | Coparenting relationship (rater NS) | 1- & 6- months PP | Yes – better coparenting relationships (1- & 6-months PP) |
| Huebner et al. (2000), USA. ‘Dialogic Reading’ | 129 parent-child dyads | PSESD | Postnatal | Public libraries | Children's librarians | Parent’s use of interactive behaviours that characterise dialogic reading (researcher) | Baseline, after sessions 1 and 2, and 3 – 4.5 months post intervention | Yes – increase in frequency of dialogic reading |
| Hyczko et al. (2021), USA. ‘INSIGHT Responsive Parenting Intervention’ | 232 mother-child dyads | PSESD | Postnatal | Home, clinical research centre,  phone | Research nurses | Infant feeding style: pressure (to finish, to soothe, with cereal), restrictive (amount, quality), responsiveness to child cues of satiety (mother)  Controlling feeding practices: pressure to eat, restriction (mother)  Child feeding: monitoring, pressure, restriction (mother) | 28 weeks PP  1-, 2- & 3-years PP  3 years PP | Yes – lower pressure to finish, pressure to soothe & pressure with cereal  Yes - lower pressure to eat  Yes - less restriction by mothers of girls than mothers of boys (intervention group only) |
| Jeong et al. (2024), Tanzania. ‘Engaging Fathers for Effective Child Nutrition and Development in Tanzania’ | 960 households | PSESD | Postnatal | Peer groups (location decided by participants), home visits | Voluntary government community health workers | Parenting: engagement in stimulation, harsh disciplinary practices (mother, father)  Perceptions of the coparenting relationship (mother, father)  Mother-child dyadic interaction (observer)  Father-child dyadic interaction (observer)  Responsive feeding practices (mother) | Baseline (pre-intervention), Midline (5 months after intervention implementation), endline (2 months post intervention) | Yes –improvements in maternal and paternal stimulation (post-intervention).  No differences on harsh disciplinary practices.  Yes – improved perceptions of coparenting relationship (fathers only; post-intervention)  No  No  No |
| Johnston et al. (2006), USA & Canada. Compared ‘Healthy Steps’ with/without ‘PrePare’  component | 343 mothers | PSESD | Both | NS | Trained facilitators with backgrounds in nursing, clinical social work or mental health practice | Nurturing parenting style (mother) | Baseline & 30 months PP | No |
| Jones et al. (2018), USA. ‘Family Foundations’ | 302 couples | PSESD, CR | Both | Health care facilities | Mixed-gender pair of trained group leaders | Co-parenting: positivity, negativity (researcher)  Parenting: positive, negative (researcher)  Triadic relationship quality (researcher)  Negative couple communication | 2 years PP | Yes - lower  co-parenting negativity  Yes - lower negative parenting  Yes - higher triadic relationship quality  Intervention impact was larger for families that had higher levels of negative couple communication at baseline |
| Kahraman et al. (2023), Turkey. Continuous intrapartum supportive care | 59 mothers | NC | Both | Hospital | Research midwife | Maternal attachment (mother) | 6-8 weeks PP | Yes – higher maternal attachment |
| Kan et al. (2015), USA^1^. ‘Family Foundations’ | 167 couples | PSESD, CR | Both | NS | Trained male-female co-leader team | Co-parenting: agreement, parent-based closeness, exposure to conflict, endorsement of partner's parenting, support, undermining (parent)  Parenting behaviour: positivity, negativity, intrusiveness, reactivity to distress (researcher) | 4 – 8 months PP  13 months PP | Yes - mothers’ & fathers’ pre-birth violence perpetration predicted lower father parenting positivity & higher father parenting negativity in control group (effects of pre-birth violence were attenuated in intervention group)  Yes - mothers’ violence perpetration negatively predicted both parents’ reactivity to distress in control group (effects of pre-birth violence were attenuated in intervention group) |
| Kaner et al. (2024), Israel. Online discussion group to support new fathers during the transition to fatherhood | 122 fathers | PSESD, CR | Postnatal | Online | Researcher | Marital quality (father) | NS | Yes – significant decline in marital quality for control group but not intervention group |
| Kavanagh et al. (2021), Australia. Compared ‘Baby Steps Wellbeing’ and ‘Baby Care’ | 248 couples | PSESD, CR, PCSS | Both | Online | Self-guided | Couple relationship satisfaction (mother, father) | Baseline (3^rd^ trimester), 13- & 26- weeks after baseline | Yes – less decline in relationship satisfaction over time in Baby Steps Wellbeing group than Baby Care group |
| Kawahara et al. (2023), Japan. Educational video promoting paternal involvement in childcare | 451 families | NC | Postnatal | Not specified | Self-guided | Paternal involvement in childcare: feeding, diaper change, bathing, changing clothes, putting child to sleep, taking child for walks, playing with child at home (mother) | 3-, 6-, 12- & 18-months PP | Yes – fathers more likely to take their children for walks  No - for all other aspects of childcare |
| Kennedy et al. (2023), England. Brief couple-focused psychotherapeutic intervention | 60 couples | CR | Both | Clinic, home | Highly experienced senior couple psychotherapists | Couple relationship: dyadic adjustment, consensus, satisfaction, cohesion, affectional expression (parent)  Couple conflict tactics (rated for self and partner): negotiation, physical assault, psychological aggression (parent) | 28 weeks G, 6-8 weeks PP, 6 months PP | No |
| Khodakarami et al. (2018), Iran. Life skills counselling | 64 mothers | CR | Prenatal | NS | Researcher with postgraduate qualifications in midwifery counselling | Marital satisfaction (mother) | Pre-intervention, post-intervention, 4 weeks post- intervention | Yes – higher marital satisfaction |
| King et al. (2023), USA. Teaching baby or learning from baby | 66 mother-child dyads | PCI | Postnatal | Laboratory | Researcher | Maternal caregiving behaviour: intrusiveness, sensitivity, cognitive stimulation, positive regard/warmth, negative regard, detachment/  disengagement (researcher) | After delivery of experimental condition instructions | Yes - higher cognitive stimulation and higher intrusiveness among mothers instructed to teach their infant  Lower warmth among mothers instructed to learn from their infant (unexpected finding)  No group differences on sensitivity, negative regard, detachment  /disengagement |
| Koushede et al. (2017), Denmark. Compared antenatal small-class education and auditorium-based lectures | 1,348 mothers | PSESD, CR, PCSS | Both | Local midwifery sites | Midwives,  health visitors | Parenting alliance (mother) | 6 months PP | No |
| Kristensen et al. (2020), Denmark. ‘Newborn Behavioural Observations’ |  | PCI | Postnatal | Home | Health visitors | Mother-infant interaction (mother) | 1-2 weeks, 3 months & 9 months PP | No |
| Leitch et al. (1999), Canada. Infant communication education (based on ‘Keys to Caregiving’) | 29 mother-child dyads | PSESD, CR, PCSS | Prenatal | NS | Self-taught (videotape) | Mother-infant interaction: caregiver, child, contingency, overall scores (researcher) | 24 hrs PP | Yes – higher total score on mother-child interaction &  higher contingency subscale scores for sensitivity to cues & social- emotional growth- fostering behaviour |
| LoCasale-Crouch et al. (2023), USA. Effective Classroom Interactions for Toddler Educators | 34 toddler educators | NC | Postnatal | Online | Instructors | Quality of child-educator interactions: emotional and behavioural support, engaged support for learning  Teaching practices to prevent/reduce challenging behaviours:  emotional guidance, behavioural and engagement guidance, peer interactions, total score | Post-intervention | Yes – higher quality child-educator interactions  (emotional and behavioural support, engaged support for learning)  Yes – higher use of effective teaching practices to prevent/reduce challenging behaviours  (emotional guidance, behavioural and engagement guidance, peer interactions, total score) |
| Lukowski et al. (2015), China. Diaper manipulation | 82 mother-child dyads | NC | Postnatal | Home, study site (NS) | Recruitment agency,  study personnel | Mother- infant interaction: infant’s engagement and positive affect, mother’s engagement and sensitivity, detachment and flat affect, negative regard for child (researcher) | Baseline, 6 weeks after intervention commenced | Yes - higher infant engagement & positive affect, higher maternal engagement & sensitivity |
| Magill-Evans et al. (2007), Canada. Video self-modelling with feedback | 162 father-child dyads | PCI | Postnatal | Home | Trained, experienced home visitors who understood infant development | Parent-child interaction: parent’s sensitivity to cues, response to distress, social-emotional growth-fostering,  cognitive growth- fostering (researcher) | 5- & 8- months PP | Yes - intervention group maintained sensitivity to infant cues, control group decreased in sensitivity. Intervention fathers improved in fostering cognitive growth |
| Matthey et al. (2004), Australia. ‘Preparation for Parenthood’ program with added ‘empathy’ session | 199 couples | PSESD, CR | Both | Hospital | Clinical psychologist and either a female  social worker/  occupational therapist experienced in counselling new parents | Partner awareness (mother, father)  Partner emotional support  (mother, father)  Sharing of infant-care tasks  (mother, father) | 6 weeks PP  Pregnancy, 6 weeks & 6 months PP  6 weeks & 6 months PP | Yes – higher partner awareness (fathers)  No  Yes - more satisfaction with sharing of infant-care tasks at 6 weeks PP (mothers with low self-esteem) |
| Metgud et al. (2023), India. ‘Sit Down and Play’ | 52 parent-child dyads | PCI | Postnatal | Hospital well baby clinic | Sit Down and Play administrators (physical therapists) | Caregiver teaching and play activities (parent)  Verbal interaction between caregiver and child (parent) | 12 months PP | Yes – improvement in parent-child stimulation activities  Yes – improvement in parent-child verbal interactions |
| Midmer et al. (1995), Canada^1^. Prenatal parenting education | 70 couples | PSESD, CR | Prenatal | Hospital | Social workers | Couple relationship: dyadic adjustment, consensus, satisfaction, cohesion, affectional expression (parent) | 2^nd^ trimester, 6 weeks & 6 months PP | Yes- higher dyadic adjustment, consensus (at 6 weeks PP), & cohesion (at 6 months PP) |
| Miller et al. (2025), USA. ‘Smart Beginnings’ (includes ‘PlayReadVIP’ and ‘Family Check Up’) | 327 mother-infant dyads | PCI, PSESD | Postnatal | Well-baby clinic (PlayReadVIP)  Home (Family Check Up) | A non-clinical Bachelor’s-level interventionist (PlayRead VIP)  Master’s level interventionist (Family Check Up) | Cognitive stimulation in the home (mother, observer)  Maternal feeding styles: responsive to satiety, pressuring to finish, indulgent permissive, indulgent soothing (mother) | 6 months PP  24 months PP | Yes – improved cognitive stimulation in the home  Yes – less likely to exhibit a pressuring feeding style  Intervention also had indirect effects on parent feeding styles (higher responsive and lower indulgent styles) via improvements in cognitive stimulation (24 months) |
| Missler et al. (2020), The Netherlands.  Psychoeducational intervention | 138 mothers and 96 partners | PCI, PSESD | Both | Home | Clinical and Developmental psychologist | Parent-infant bonding (parent) | 6 - & 10-weeks PP | No |
| Moreno et al. (2015), USA. ‘Expanding Quality for Infants and Toddlers (EQ)’ | 183 caregivers | NC | Postnatal | Classroom, workplace/outside classroom | EQ instructors/coaches with qualifications in education or human development related fields | Classroom: emotional-behavioural support, support for language and learning (researcher) | Pre-test, post-test (4-6 months later), follow-up (4 months after post-test) | Yes- increased support for learning and language at follow-up (largest in EQ15 group) |
| Murray et al. (2023), UK. Dialogic book-sharing | 218 families | PCI | Postnatal | Children's centres | Trained research facilitators with a background of working in the early years context. | Parent behaviour: sensitivity, cognitive scaffolding, mental state talk (researcher)  Parent-child interaction: general reciprocity, verbal reciprocity (researcher) | Baseline, post-intervention, and 4-6 months post-intervention | Yes – increases for all outcomes post-intervention |
| Murray-Perdue et al. (2024), USA | 202 couples | PSESD, CR | Postnatal | Home | Trained coaches | Maternal and paternal spousal romantic attachment (mother, father)  Maternal and paternal constructive conflict behaviour (researcher) | 6-months PP (pretest) & 12-, 16- & 18 months PP (follow-ups) | Yes- lower maternal attachment security at 18 months PP (unexpected finding; received conflict intervention only)  Yes – higher paternal constructiveness at 18 months PP (received conflict intervention only) |
| Myers et al. (1982), USA^1^. Brazelton intervention | 42 parent-child dyads | PCI | Postnatal | Hospital | Researcher | Mother-infant interactions, father-infant interactions (researcher)  Father’s caretaking (father)  Satisfaction with infant (parent)  Satisfaction /  affection with infant: first felt love, first felt baby was theirs, (parent) | 1 day PP  4 weeks PP  1 day PP  4 weeks PP | No  Yes - higher father involvement in infant caretaking  Yes - higher parent satisfaction with infant at 1 day PP  No |
| Niccols et al. (2008), Canada^1^. ‘Right from the Start’ | 76 mothers | PSESD, PCSS | Postnatal | Convenient locations (NS) | Infant development specialists with backgrounds in psychology, early childhood education, and/or social work. | Infant attachment security (researcher)  Mother’s sensitivity (researcher) | Baseline, post-intervention, 6-month follow-up  Baseline, post-intervention, 6-month follow-up | No  *When intervention attendance was accounted for, attendees in both groups showed higher maternal sensitivity & infant attachment security than non-attendees |
| Niccols et al. (2009), Canada. ‘COPEing with Toddler Behaviour’ | 79 mothers | PSESD, PCSS | Postnatal | Early years centres | Infant development specialists with backgrounds in psychology, early childhood education, and/or social work. | Parenting behaviour: laxness, overactivity (mother)  Parent-child interaction: child-positive, child – negative, mother-positive, dyad – compliance (researcher) | Baseline, post-intervention, 1 month follow-up  Baseline, post-intervention, 1 month follow-up | Yes – lower mother overactivity  Yes - higher positive child behaviour (1 month follow-up), positive mother behaviour (post-test, 1 month follow-up) & dyad compliance (post-test, 1 month follow-up) |
| Oles et al. (2024), Honduras. ‘Proyecto Redes: Con Amor y Cuidados Madres y Bebés Sanos (With Love and Care, Healthy Moms and Babies)’ | 263 parents | PSESD | Postnatal | Home | Community Change Agents (community health workers) | Paternal involvement: father held child, father played with child (parent) | Post-intervention | No |
| Park et al. (2025), South Korea. Mindfulness-based mobile intervention (‘AvecMom’) | 94 mothers | PSESD | Prenatal | Online (via app) | Self-guided | Maternal-fetal attachment: differentiation of self from fetus, interaction with the fetus, attributing characteristics to the fetus, giving of self, maternal role-taking (mother) | Pre-test, post-intervention | Improvements on maternal-fetal attachment: attributing characteristics to fetus &  differentiating self from fetus (post-intervention) |
| Pontoppidan et al. (2016), Denmark. ‘Incredible Years Parents and Babies Program’ | 104 parent-child dyads | PSESD | Postnatal | NS | Trained group leaders | Mother-child interaction (mother) | Baseline, 1-3 weeks  post-intervention | No |
| Popp et al. (2019), Germany. ‘Baby Triple P - Positive Parenting Program’ | 49 parents | PSESD | Both | Face to face (NS), phone | Trained clinical psychologists | Quality of partnership (parent) | Before birth, 10 weeks & 6 months PP | No |
| Prado et al. (2017), Malawi. Maternal supplementation | 669 mothers | NC | Prenatal | Home | Research staff, research nurses | Mother’s caregiving (researcher, mother) | 6 months PP | No |
| Reilly (2025), USA. Loving-Kindness Meditation | 136 parents | PSESD | Postnatal | Online | Researcher | Parent sensitivity (researcher) | Post-intervention | No |
| Rheinheimer et al. (2022), The Netherlands. Skin-to-skin contact | 116 mother-child dyads | PCI | Both | Home | Researcher | Quality of mother’s caregiving (researcher)  Mother-infant adrenocortical synchrony (cortisol samples) | 5 weeks PP | No |
| Robertson et al. (2018), USA. Contingent lullaby music | 45 mother-child dyads | PCI | Postnatal | Medical centres, birthing centre | Researcher | Parent-infant interaction; mother’s responsiveness, warmth (researcher)  Frequency of interaction behaviour: touching, looking, talking, smiling, infant response, and singing  (researcher) | 6 weeks PP | Yes – higher mother responsiveness & warmth  Yes - more frequent mother-infant eye contact, mother-infant talking, infant response, & singing |
| Ruggiero et al. (2021), USA. ‘INSIGHT Responsive Parenting Intervention’ | 232 mother-child dyads | PSESD | Postnatal | Home | Research nurses | Parent feeding practices: restriction, pressure, food to soothe, food as a reward (mother) | 3 years PP | Yes - less pressure to eat, food to soothe, & food as a reward |
| Sabancı Baransel (2023), Turkey. Attachment-based intervention program | 154 mothers |  | Prenatal | Hospital outpatient clinics | Researchers | Maternal antenatal attachment: quality of attachment, time spent on attachment, total score (mother) | Pre- & post-test (days 7 later) | Yes – increased quality of attachment, time spent on attachment, and overall attachment |
| Sandborg et al. (2025), Australia. ‘Let’s Grow’ app | 1165 parents | PSESD, PCSS | Postnatal | Online (via app) | Self-guided | Parental behaviours – coparticipation (parent)  Parental modelling (parent)  Parental behaviours: family rules, family routines (parent) | Baseline, 6-month follow-up | No |
| Santelices et al. (2010), Chile. ‘Promoting Secure Attachment’ | 72 mother-child dyads | PCI | Both | Health centres | Researchers | Infant attachment (researcher) | Post intervention (during first year PP) | No |
| Sari Ozturk et al. (2023), Turkey. Mandala activity and technology-based breastfeeding program | 66 mother-child dyads | PCI | Both | Hospital | Researchers | Mother-infant attachment (mother) | 1 week & 2 months PP | Yes – higher attachment |
| Savage et al. (2018), USA. ‘INSIGHT Responsive Parenting Intervention’ | 279 mother-child dyads | PSESD | Postnatal | Home | Research nurses | Parent feeding practices; feeding to soothe - emotional, contextual (mother)  Infant feeding style: pressure (to finish, to soothe, with cereal), restrictive (amount, diet quality) responsive satiety (mother) | 8-,16-, 32- and 44-weeks PP  28 weeks PP | Yes – less frequent use of feeding to soothe in response to emotional or contextual factors  Yes - lower pressure to finish & use of feeding to soothe |
| Schroeder et al. (2015), USA. ‘Growing Leaps and Bounds’ | 232 families | PSESD | Postnatal | Health centres | Paediatricians, nurse practitioners, clinic staff | Child feeding practices – dietary restriction, pressure to eat, and monitoring (parent) | 12- & 24-months PP | Yes – more dietary restriction & more active in monitoring child feeding (both at 24 months) |
| Selkirk et al. (2006), Australia. Midwife-led postnatal debriefing | 143 mothers | PSESD | Postnatal | Hospital | Hospital midwives | Couple dyadic adjustment (mother) | 3^rd^ trimester, 3 months PP | No |
| Setodeh et al. (2018), Iran. Paternal-attachment training skills | 150 couples | PCI, PSESD | Prenatal | NS | Researchers | Mother-neonatal attachment (researcher) | 24 hours PP | Yes – higher attachment |
| Shapiro et al. (2011), USA. ‘Bringing Baby Home’ | 181 couples | PSESD, CR | Both | Hospital | Birth preparation teachers | Mother-father-infant triadic interaction: mother engaged and competitive, father engaged and competitive, calm and complementary play, parents coordinated and baby disengaged (researcher) | 3 months PP | Yes – rated lower on ‘father engaged & competitive’, & ‘mother engaged & competitive’ |
| Shapiro et al. (2020), USA. ‘Bringing Baby Home’ | 119 couples | PSESD, CR | Both | Hospital | Birth preparation teachers | Father’s involvement in parenting tasks (parent) | 3 months PP | Yes – more equal distribution of parenting tasks |
| Shariat et al. (2017), Iran. Psychological intervention | 71 mothers | PSESD | Prenatal | Maternity clinics | Clinical psychologist | Mother-infant attachment, (mother) | Baseline, 1-, 3- & 7- days PP, 1-, 3-, 6-, 8 & 12- months PP | Yes – larger increase in attachment |
| Shen et al. (2021), Taiwan. Non-supervised aerobic exercise intervention | 123 mothers | PCI | Prenatal | Home | DVD of a prenatal yoga teacher | Mother’s attachment to foetus (mother) | 1- & 3-months post intervention | Yes – higher attachment |
| Shorey et al. (2023), Singapore. ‘Supportive Parenting App’ | 200 couples | PCSS | Both | Hospital, home | Mobile app, peer volunteers | Parent-infant bonding (mother) | Baseline, 1-, 2, - 4-, 6-, 9- & 12 months PP | Yes - higher parent-infant bonding in control group at 6 months PP (unexpected finding) |
| Shorey et al. (2017), Singapore. ‘Home-but not Alone’ | 125 couples | PSESD, PCSS | Postnatal | Home | Research assistant, mobile app, clinical staff, peers | Perceived social support from partner (parent) | Baseline, 4 weeks PP | Yes – higher social support from partner |
| Shorey et al. (2019), Singapore. ‘Supportive Educational Parenting Program’ | 118 couples | PCSS | Both | Hospital | Research assistant, mobile app | Parent-infant bonding (mother) | Baseline, immediate PP, 1- & 3 months PP | Yes – higher parent-infant bonding (1-&3-months PP) |
| Smith et al. (2023), USA. ‘Music Together’ | 75 parent-child dyads | PCI | Postnatal | Local music studio | Researcher | Mother-child relationship: mother’s warmth/sensitivity, negative affect/harshness, intrusiveness (researcher) | Baseline, 6, 12, 24 months post-intervention | Decreased mother harshness (from baseline to 12 months & 6 months to 12 months) & mother intrusiveness (between 6-12 months) |
| Solmeyer et al. (2014), USA. ‘Family Foundations’ | 132 couples | PSESD | Both | Health care facilities | Trained male-female co-leader team | Coparenting positivity: warmth and cooperation (researcher)  Child adjustment problems | 1 year PP  3 years PP | No  Yes – competition in coparenting mediated intervention effects on child adjustment problems for fathers (for sons & daughters) & mothers (for sons only) |
| Sönmez et al. (2025), Turkey. Hybrid breastfeeding counselling | 90 mothers | PSESD | Both | Hospital, online | Researcher | Mother-infant bonding | 1-5 days PP (pre-test) & 13-24 weeks PP (post-test) | Yes - improved mother-infant bonding (post-test) |
| Sosa et al. (1980), Guatemala. Supportive birth companion | 40 mothers | PCSS | Prenatal | Hospital | Trained support companions | Mother-infant interaction: handling of infant, any verbal or nonverbal communication with infant & nursing behaviours (researcher) | Immediately PP | Yes – more frequent stroking and talking to infants |
| Suchy et al. (2020), USA. Infant massage instruction | 98 fathers | PCI | Postnatal | Hospital, community health centre | Nurses, certified massage instructor | Father-infant interaction: verbal interaction, smiling, eye contact,  fingertip touching, whole-hand touching, total score (researcher) | 12 – 48 hours PP | Yes – more total interactions & fingertip touching in fathers observed immediately after watching video, but less verbal interaction (unexpected finding) in this group |
| Taylor et al. (1985), USA. Extra physical contact immediately after birth | 78 mothers- child dyads | PCI | Postnatal | Hospital, home | Nursing staff | Mother-infant interaction (researcher) | 2 days & 1 month PP | No |
| Toney et al. (1983), USA. Father holding newborn post-delivery | 37 father-child dyads | PCI | Postnatal | Hospital | Graduate nursing students | Father-infant interaction: verbal interaction, smiling, eye contact,  fingertip touching, whole-hand touching, (researcher) | 12 – 36 hours PP | No |
| Turk Dudukcu et al. (2022), Turkey. Health promotion program | 64 mother-child dyads | PSESD | Both | Family health centre | Researchers | Mother’s attachment (mother) | 36 weeks G, 8 - & 24weeks PP | Yes - higher mother’s attachment (8- & 24- weeks PP) |
| Uçakcı et al. (2023), Turkey. Online health training/counselling and ‘Progressive Muscle Relaxation Exercise (PMRE)’ | 52 mothers | PSESD | Both | Home via videocall | Researcher | Maternal attachment | 6 weeks PP | Yes – higher maternal attachment |
| Uncu et al. (2025a), Turkey. Father birth attendance | 103 fathers | NC | Both | Hospital | Researcher | Paternal attachment (father) | 2 weeks PP | Yes – higher paternal attachment |
| Uncu et al. (2025b), Turkey. Newborn massage training | 54 mothers | PCI | Both | Online | Researcher | Maternal attachment (mother) | 6- &10-weeks PP | Yes – higher maternal attachment |
| van Vliet et al. (2022), The Netherlands. ‘Video intervention to promote positive parenting–feeding infants’ | 246 mother-child dyads | PCI | Postnatal | Home | Researchers | Mother’s responsiveness to satiety cues  (researcher)  Mother’s sensitivity (researcher)  Mother’s pressure to eat  (researcher)  Mother’s responsive feeding (mother)  Mother’s pressure to eat (mother) | Baseline, 18 months PP, 24 months PP  Baseline, 18 months PP, 24 months PP  18 months PP, 24 months PP  18 months PP, 24 months PP  18 months PP, 24 months PP | Yes–higher responsivity to satiety cues in VIPP and COMBI groups than AC group (18 months PP)  No  No  Yes - higher responsive feeding in COMBI group than RVE & AC groups (18 months PP)  Yes - less pressure to eat in VIPP & COMBI groups than RVE group (18 months PP) |
| Verhees et al. (2023), The Netherlands. Baby carrier for fathers | 80 fathers | PCI | Postnatal | Home | Researcher | Paternal sensitivity (researcher)  Paternal involvement (father) | Baseline and post-intervention | No |
| Vlasblom et al. (2020), The Netherlands. ‘BBOFT+’ program | 1,195 parent-child dyads | PSESD | Postnatal | Well-baby clinics | Community physicians, nurses | Parenting style: parental warmth, control (parent)  Parenting practices relating to eating and activity - limit setting, control, monitoring, discipline, reinforcement (parent) | 14- & 36- months PP (warmth), 36 months PP (control)    36 months PP | Yes – higher parental control |
| Waldenström et al. (1999), Sweden Birth centre care | 1143 fathers | PSESD | Both | Hospital | Midwives | Father involvement in birth process (father)  Impact of birth attendance on partner relationship (father)  Impact of birth attendance on immediate feelings for newborn (father)  Father home from work within 2 weeks of birth, number of days home (father)  Father taking/already planned additional parental leave, total planned parental leave (months)  Changed diapers during the last 24h, number of diapers changed (father)  Bathed or washed baby during the last 24h (father)  Total time (in hours) spent with baby during the last 24h (father) | 2 months PP | No  No  No  Yes – father spent more days at home during first two weeks  No  No  No  No |
| Walter et al. (2019), Germany. ‘SAFE – Secure Attachment Family Education’ | 104 mothers, 77 fathers | PSESD, PCSS | Both | Hospital | Trained health and social-service professionals | Infant-father attachment (researcher)  Infant-mother attachment (researcher) | 18 months PP | Yes – higher % of infants were securely attached to their fathers  No |
| Weiland et al. (2023), USA. ‘Nurture®’ program | 201 mothers | PSESD, PCSS | Both | Researcher and mentors (mostly teachers, nurses or social workers) | Home | Paternal involvement (mother) | 4-& 18-months follow-up | No |
| Westerneng et al. (2022), The Netherlands. Third trimester routine ultrasound | 1,275 mothers | PCI | Prenatal | Primary care midwifery practices, ultrasound centres, hospitals | Sonographers | Mother’s bonding to foetus (mother) | between 20- & 27-weeks' G, 32 weeks' G | Yes- higher bonding (intervention mothers with low bonding at baseline) |
| Wigglesworth et al. (2023), England. Sling provision and training | 55 mother-child dyads | PCI, PSESD, PCSS | Postnatal | Peer volunteers and trained staff | Sling library | Mother-infant relationship | 0-6 weeks PP (baseline), 12 weeks post-baseline | No |
| Witte et al. (2022), The Netherlands. Intranasal administration of oxytocin and vasopressin | 70 father-child dyads | NC | Postnatal | NS | Researchers | Father’s sensitivity (researcher)  Challenging parenting behaviours (with and without toys) (researcher) | After hormone administration (2-12 months PP) | Yes - lower paternal sensitivity with oxytocin (unexpected finding)  No |
| Witte et al. (2024), The Netherlands. Baby carrier for fathers | 80 fathers | PCI | Postnatal | Researcher | Home | Language and early literacy practices (father) | Baseline, post-intervention, & 5 months post-intervention | No |
| Wulff et al. (2021a), Germany. Prenatal music intervention | 172 mothers | PCI | Prenatal | Hospital, home | Music therapist | Mother’s antenatal attachment (mother)  Perceived closeness to infant (mother) | Pre-intervention, post-intervention (36th weeks G) | No  Yes – largest increase in perceived closeness with infant in singing group |
| Wulff et al. (2021b), Germany. Mother-infant singing intervention | 120 mother-child dyads | PCI | Postnatal | Hospital, home | Music therapist | Mother-infant attachment (mother)  Perceived closeness to infant(mother) | 48 hours PP (baseline), 2 weeks after baseline (T1), immediately pre- and post- intervention, 12 weeks PP (T2) | No  Yes – increase in perceived closeness to infant between pre- and post- intervention |
| Yildirim et al. (2023), Turkey. ‘Kangaroo Care’ | 90 fathers | PCI | Postnatal | Researcher | Hospital | Father-infant attachment (father) | 3 months PP | Yes – higher father-infant attachment |
| Yildirim et al. (2025), Turkey. Infant care training for new fathers | 63 fathers | PSESD | Postnatal | Hospital, phone call | NS | Father-infant attachment (father)  Baby care participation (father) | 3 months PP  2-, 3-, 4-, 8- & 12 weeks PP | Yes- higher father-infant attachment  Yes – higher participation in baby care |
| Zachariah Boukydis et al. (2006), USA. Prenatal ultrasound consultation | 52 mothers | PCI | Prenatal | Hospital | Sonographer | Mother’s attachment to foetus: overall score and attributing characteristics to foetus (mother) | Immediately before and after ultrasound examination | Yes- increase in both outcomes |

Notes: PCI = Parent-child interaction. CR = Couple relationship. PSESD = Parental support, education and skill development.

PCSS = Parental community and social support. NC = Not classified. NS=Not specified. PP= Post partum. G = Gestation. ^1^based on author’s affiliation. ^2^Sig group differences are with respect to the control group and in expected direction unless otherwise stated. Statistical significance threshold is p<0.05.
